# Structural, optical, and magnetic characterization of Cu–Zn–Ni spinel ferrite nanoparticles with antibacterial potential

**DOI:** 10.1038/s41598-025-34792-9

**Published:** 2026-01-22

**Authors:** Samaa Ali, O. M. Hemeda, F. Elhussiny, M. H. Mahgoub, Sh. Mohammed, Ahmed Elmekawy

**Affiliations:** 1 Physics Department, Faculty of Science, Tanta University, Tanta, 31527 Egypt; 2 Botany and Microbiology Department, Faculty of Science, Tanta University, Tanta, 31527 Egypt

**Keywords:** Antibacterial, Ferrite nanoparticle, *Escherichia coli*, *Staphylococcus aureus*, Gentamycin, Chemistry, Materials science, Microbiology, Nanoscience and technology

## Abstract

One of the most pressing challenges in biomedical applications is the growing prevalence of bacteria that are resistant to multiple antibiotics. Metal-based nanoparticles are emerging as a promising strategy to address this problem, which is the focus of the present work. Cu_0.15_Zn_0.2_Ni_0.65_Fe_2_O_4_ nano-ferrite was synthesized via the co-precipitation method. The chosen cation ratio preserves the spinel phase while Ni improves magnetic response, and Zn enhances magnetic softness and site stability. For comparison, single-cation ferrites NiFe_2_O_4_, ZnFe_2_O_4_, and CuFe_2_O_4_ were synthesized using the same procedure to enable a consistent evaluation of antibacterial activity. All ferrites were characterized using XRD and FTIR. Additional analyses including UV–Vis, SEM, EDX, XPS, TEM, VSM, and Atomic Absorption Spectroscopy (AAS) were performed for Cu_0.15_Zn_0.2_Ni_0.65_Fe_2_O_4_ sample. XRD confirmed a cubic spinel phase for all ferrites. FTIR provided further evidence of cation redistribution of tetrahedral and octahedral sites. AAS verified the availability of Cu^2+^, Zn^2+^, and Ni^2+^ ions, supporting their contribution to antibacterial activity. VSM showed soft magnetic behavior with ~ 54.3 emu/g saturation magnetization. Antibacterial tests demonstrated that Cu_0.15_Zn_0.2_Ni_0.65_Fe_2_O_4_ exhibits stronger inhibitory activity against *S. aureus* and *E. coli* at both low and high concentrations. At 500 μg/mL, the inhibition zone reached ~ 20 mm for *S. aureus* and ~ 17 mm for *E. coli*, The MIC values were found to be 40 μg/mL for *S. aureus* and 80 μg/mL for *E. coli*, indicating stronger sensitivity of Gram-positive bacteria. After establishing its individual performance, comparison has been obtained with single-cation ferrites. Across all trials, Cu_0.15_Zn_0.2_Ni_0.65_Fe_2_O_4_ consistently produced larger inhibition zones, showing clear superiority. The superior antibacterial activity is attributed to the synergistic incorporation of Cu^2+^, Zn^2+^, and Ni^2+^ within a single spinel lattice, giving Cu_0.15_Zn_0.2_Ni_0.65_Fe_2_O_4_ strong intrinsic antibacterial activity and improving performance over single-cation ferrites, confirming its novelty and potential for biomedical applications.

**Supplementary Information:**

The online version contains supplementary material available at 10.1038/s41598-025-34792-9.

## Introduction

Magnetic spinel nanoferrites are an important class of nanomaterials with the general formula MFe_2_O_4_, where M denotes a divalent metal ion that may belong to transition, alkaline earth, post-transition, or rare earth elements. They are distinguished by their remarkable structural, chemical, and magnetic properties^1^, which can be tailored through the selective substitution of cations (e.g., Mg^2+^, Zn^2+^, Cu^2+^). Such substitutions markedly influence their magnetization, magnetic permeability, and hysteresis characteristics^2–4^. These features have enabled their integration into diverse technological sectors, including data storage, electronic and communication systems, catalysis, sensing technologies, and environmental monitoring^5–16^. Additionally, their biocompatibility, combined with the ease of surface functionalization, further enhances their suitability for biomedical applications, such as hyperthermia treatment^5^, targeted drug delivery^17^, bioimaging, and biosensing^18^.

**Fig. 1:**
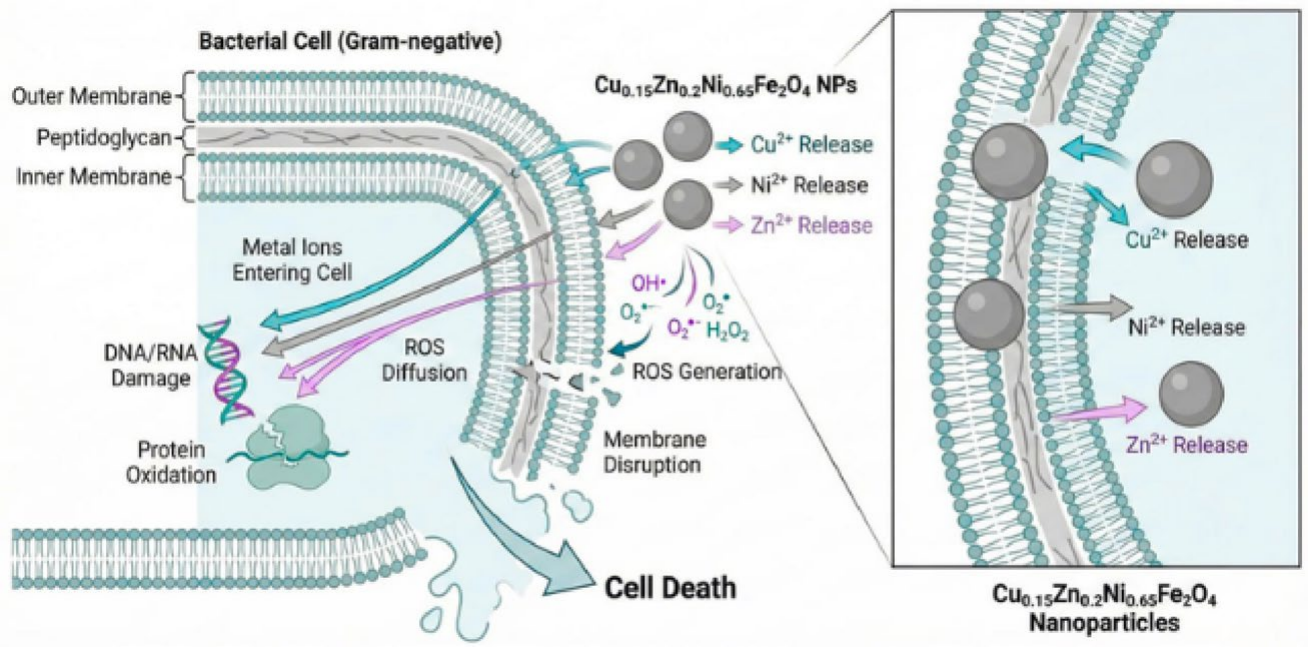
Schematic illustration the antibacterial mechanism of Cu_0.15_Zn_0.2_Ni_0.65_Fe_2_O_4_ nanoparticles.

In biomedical applications, antibacterial functionality is particularly important for bone-related therapies and medical implants, where postoperative infections such as osteomyelitis remain a serious complication^19^. These infections can lead to severe inflammation and bone resorption, while treatment is often hindered by the limited vascularization of bone tissue. As a result, high systemic doses of antibiotics are typically required, which may cause adverse effects on organs such as the liver and kidneys. Nanoparticles are increasingly employed as alternatives to conventional antibiotics due to their multifaceted antibacterial behavior. Their antimicrobial action generally proceeds through three primary pathways as Shawn in Fig. 1: (i) direct physical interaction with bacterial cells, (ii) the release of metal ions, and (iii) the generation of reactive oxygen species (ROS)^20–22^. In the first pathway, nanoparticles with its small size adhere to and disrupt the bacterial cell membrane, enabling their penetration into the cell and causing leakage of intracellular components. This disruption leads to the degradation of essential biomolecules, including proteins, DNA, and enzymes^23,24^. The second antibacterial mechanism involves the release of metal ions from ferrite materials through their gradual dissolution, a process strongly influenced by environmental factors conditions. Smaller nanoparticles often release ions at rates that differ from those of larger ferrite particles due to variations in surface area and dissolution behavior. Ion release generally begins with the breakdown of the ferrite crystal lattice through dissolution or leaching. Once released, these ions can interact with amino (–NH), thiol (–SH), and carboxyl (–COOH) functional groups in macromolecules such as proteins and nucleic acids. These interactions may inhibit enzymatic activity, induce structural alterations, and ultimately disrupt normal cellular physiological functions^20,25,26^. A third antibacterial mechanism is associated with ROS generation, the positive surface charge of the nanoparticles facilitates electrostatic attraction to the negatively charged bacterial envelope, enhancing membrane contact. This interaction promotes local production of reactive oxygen species (ROS), which can oxidize membrane lipids, disrupt proteins, and induce DNA damage^21^. For this, ion-releasing antibacterial nanomaterials have emerged as an attractive approach for localized infection control, with cation substitution providing additional advantages.

Azam et al. (2012) demonstrated that the antibacterial activity of CuO nanoparticles is size-dependent, with smaller particles exhibiting significantly stronger effects against both Gram-positive and Gram-negative bacteria^27^. Applerot et al. (2012) reported that reduced particle size enhances antimicrobial performance, attributing this effect to the higher ability of smaller CuO nanoparticles to penetrate bacterial cells^28^. Thekkae Padil and Černík (2013) synthesized CuO nanoparticles via a colloid-thermal method and confirmed that smaller particles (4.8 ± 1.6 nm) displayed superior antibacterial efficiency compared to larger ones (7.8 ± 2.3 nm)^29^.

Zn-Ni ferrites and Cu-Ni ferrites have significant potential as antibacterial agents^30–32^. Studies have shown that they not only improve antimicrobial efficacy but also support better integration of implants with surrounding host tissues. Kurian et al. (2016) highlighted that the synthesis route and processing conditions strongly influence the structural features of Ni–Zn ferrites. In particular, samples prepared through a sol–gel route using ethylene glycol and subsequently calcined at 550 °C achieved the smallest crystallite size (17 nm)^33^. In another study, Naik et al. (2019) employed a microwave-assisted green synthesis method using wood apple juice to produce zinc ferrite nanoparticles with a cubic spinel structure and an average crystallite size of ~ 20 nm. These particles exhibited notable antibacterial activity, showing high inhibition against *E. coli* (10.50 ± 0.29 mm zone of inhibition) and *S. aureus*, while displaying moderate effects against *K. aerogenes* and *P. desmolyticum*^34^. More recently, Paz-Díaz and Garibay-Febles (2023) synthesized ZnFe_2_O_4_ and CuFe_2_O_4_ nanoparticles through a simple, cost-effective, and environmentally friendly process. Both types of nanoparticles demonstrated strong antibacterial properties, particularly against the Gram-positive bacterium *S. epidermidis*. Their findings suggest that ZnFe_2_O₄ and CuFe_2_O₄ nanoparticles hold promise as antimicrobial agents that could be incorporated into various surfaces to prevent bacterial colonization^35^. In another study, Kumar and Kandwal (2023) successfully prepared Ni–Zn–Co ferrites (Ni_0.6−x_Zn_0.4_Co_0.132_; x = 0.0264, 0.0528) which displayed strong antibacterial activity against *Bacillus subtilis* and *Escherichia coli*^36^. Samatha et al. (2024) synthesized NixCo_1−x_Fe_2_O_4_ nanoparticles (x = 0.0–1.0) using a citrate precursor sol–gel method. Their findings revealed that *E. coli* exhibited a greater inhibition zone (7 mm) than *S. aureus* (4 mm), particularly at x = 0.4. Furthermore, antibacterial activity generally increased with higher nanoparticle concentrations, reaching a maximum at 800 µg/mL^30^. Kamo et al. (2024) provided a comprehensive overview of oxide-based photocatalytic nanomaterials, including TiO_2_, ZnO, SnO_2_, CuO, and Zn_2_SnO_4_, highlighting the mechanistic pathways of their antibacterial activity and emphasizing how intrinsic nanoscale features such as size, shape, and surface charge govern their disinfection efficiency^23^. More recently, Sonmezoglu et al. (2025) demonstrated that zinc stannate nanoparticles can be synthesized in controlled phases at low temperatures using an eco-friendly reduction route, and further showed that their piezo- and flexo-phototronic properties enable highly efficient antibacterial performance across different crystallographic phases^37^.

Previous studies have consistently shown that both Ni–Zn and Cu–Ni spinel nanoferrites, when studied individually, exhibit remarkable magnetic properties and hold significant potential for biomedical applications. Their characteristics can be effectively tailored either through substitution with transition metal ions or by modifying synthesis conditions. Building on this knowledge, the present work focuses on investigating the structure, morphology, elemental composition, cation distribution, magnetic and antibacterial behavior of a multicomponent Cu–Ni–Zn ferrite with the composition Cu_0.15_Zn_0.2_Ni_0.65_Fe_2_O_4_ synthesized by co-precipitation method. For a consistent evaluation of antibacterial performance, its activity was compared with the synthesized single-cation ferrites NiFe_2_O_4_, ZnFe_2_O_4_, and CuFe_2_O_4_.

The selected stoichiometry Cu_0.15_Zn_0.2_Ni_0.65_Fe_2_O_4_ was chosen to achieve a synergistic balance between magnetic softness and antibacterial functionality. Ni and Zn are well-known for producing soft magnetic ferrites with low coercivity and high permeability, as demonstrated in studies on Ni–Zn ferrite composites^38–42^, The partial substitution of Cu enhances the antibacterial efficacy without significantly affecting the magnetic softness^13,43–45^. Thus, the specific cation ratio is optimized to preserve the cubic spinel structure (limited Cu), enhance magnetic response (high Ni), and promote magnetic softness and structural stability (Zn) to achieve favorable magnetic properties for biomedical utility with effective antibacterial activity.

## Methodology

### Ferrite synthesis procedures

**Fig. 2:**
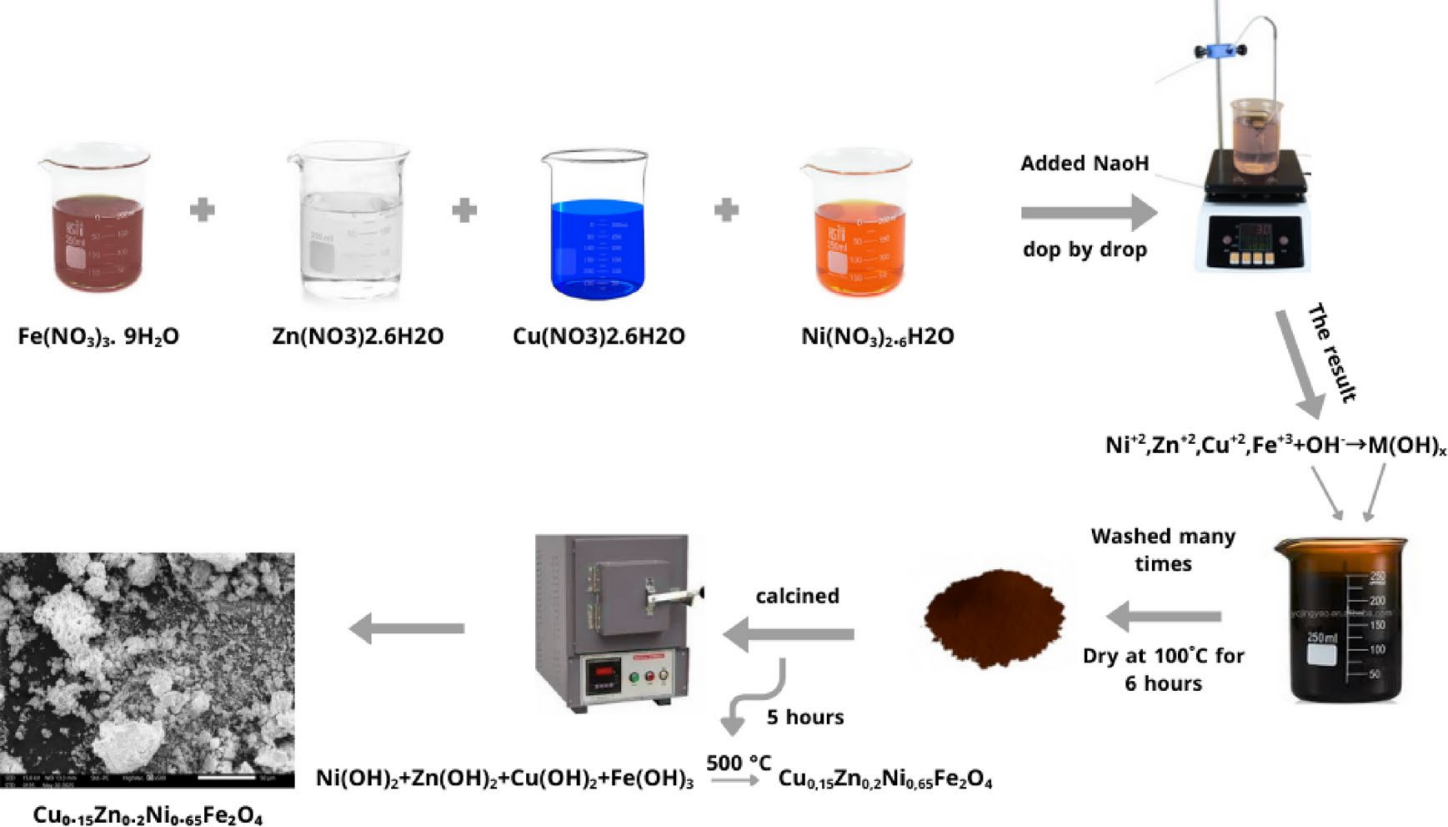
A flowchart for the preparation of Cu_0.15_Zn_0.2_Ni_0.65_Fe_2_O_4_ nano ferrite.

Cu_0.15_Zn_0.2_Ni_0.65_Fe_2_O_4_ nanoparticle was prepared through the co-precipitation technique. Stoichiometric amounts of high-purity metal nitrate salts (99.9%) were used as precursors. 3.78 g of Ni(NO_3_)_2._6H_2_O, 1.18 g of Zn(NO_3_)_2._6H_2_O, 0.72 g of Cu(NO_3_)_2_.6H_2_O, and 16.16 g of Fe(NO_3_)_3_.9H_2_O. Each salt was dissolved separately in 50 mL of distilled water. The solutions were mixed and heated at 80 °C under strong magnetic stirring (300–1000 rpm) for 2 h. The pH was adjusted to 10–12 by adding ~ 3 M NaOH dropwise as the precipitating agent. The resulting precipitate was collected, centrifuged, and washed repeatedly with distilled water to remove residual sodium salts, then dried under vacuum at 100 °C for 6 h. The dried powders were calcined at 500 °C for 5 h and left to cool naturally inside the furnace overnight to reach room temperature. Finely ground in an agate mortar to obtain homogeneous powders for further characterization. A flowchart for the preparation of Cu_0.15_Zn_0.2_Ni_0.65_Fe_2_O_4_ nano ferrite is illustrated in Fig. 2.

To enable proper comparison with single-cation spinel ferrites CuFe_2_O_4_, NiFe_2_O_4_, and ZnFe_2_O_4_ were synthesized using the same procedure and thermal conditions. For CuFe_2_O_4_, 4.83 g of Cu (NO_3_)_2._6H_2_O and 16.6 g of Fe (NO_3_)_3_·9H_2_O were each dissolved in 100 mL of distilled water. NiFe_2_O_4_ was prepared by dissolving 5.82 g of Ni(NO_3_)_2_·6H_2_O and 16.6 g of Fe(NO_3_)_3._9H_2_O in separate 100 mL aqueous solutions, while ZnFe_2_O_4_ was synthesized using 5.95 g of Zn(NO_3_)_2_·6H_2_O and 16.6 g of Fe(NO_3_)_3_·9H_2_O, also dissolved individually in 100 mL of distilled water.

### Process of antibacterial assay

The antibacterial effect of synthesized nano-ferrites was assessed toward Escherichia coli as gram-negative bacteria (*E. coli*) and *Staphylococcus aureus* as gram-positive bacteria (*S. aureus*) agar well diffusion method^46^. To perform the antibacterial assay firstly the bacterial strains were purified by culturing it on nutrient agar media and incubated at 37 °C for 24 h. The synthesized nano-ferrites will be prepared and sonicated by probe sonication in dimethyl sulfoxide (DMSO) for one hour with ice around it to prevent the heating of nano-ferrites, then *E. coli* and *S. aureus* were cultured on MacConkey agar and Blood agar respectively.

The bacterial colonies picked from purified strains were prepared in isotonic solution (0.85% NaCl) according to standard turbidity of 0.5 McFarland^46^, the concentration of the bacterial suspension standard was 1 × 107 CFU/ml.

A lawn culture from the abovementioned bacterial suspensions were made on four Muller Hinton agar (MHA) petri dishes then performed four wells in two MHA petri dishes by sterile corkborer, (i) first well poured with 50 μl of DMSO (Negative control), (ii) second well poured with 50 μl from high concentration of Cu_0.15_Zn_0.2_Ni_0.65_Fe_2_O_4_ (500 μg/ml), (iii) third well poured with 50 μl of gentamycin (4μg/ml) as Positive control, iv) fourth well with 50μl from low concentration of Cu_0.15_Zn_0.2_Ni_0.65_Fe_2_O_4_ (100μg/ml).

sixth wells were performed in others two MHA petri dishes to comparing antibacterial effect between Cu_0.15_Zn_0.2_Ni_0.65_Fe_2_O_4_ and CuFe_2_O_4_, NiFe_2_O_4_, and ZnFe_2_O_4_ nano-ferrites, (i) first well poured with 50 μl of DMSO (Negative control), (ii) second well poured with 50 μl from high concentration of Cu_0.15_Zn_0.2_Ni_0.65_Fe_2_O_4_ (500μg/ml), (iii) third well poured with 50 μl from high concentration of ZnFe_2_O_4_ (500μg/ml), (iv) fourth well poured with 50 μl of gentamycin (4 μg/ml) as (Positive control), (v), fifth well poured with 50 μl from high concentration of NiFe_2_O_4_ (500μg/ml), (vi) Sixth well poured with 50 μl from high concentration of CuFe_2_O_4_ (500μg/ml), all petri dishes were incubated at 3700 °C for 24 h after that inhibition zone around each well measured in millimeter by scale meter. All experiments were conducted in triplicate and the average values were reported^47^.

The antimicrobial activity of the synthesized Cu_0.15_Zn_0.2_Ni_0.65_Fe_2_O_4_ nanoparticles was confirmed through the appearance of inhibition zones of varying diameters (mm), against *S. aureus* and *E. coli*. The nanoparticles exhibited significant inhibitory effects on both Gram-positive and Gram-negative strains, demonstrating their broad-spectrum antimicrobial potential.

### Determination of MIC and MBC antibacterial assay

Minimum inhibitory concentration (MIC) and minimum bactericidal concentration (MBC) were determined using a standard broth micro-dilution method with minor modifications^48,49^. Serial two-fold dilutions of Cu_0.15_Zn_0.2_Ni_0.65_Fe_2_O_4_ nanoparticles (10–1280 μg/mL) were prepared in nutrient broth, and each tube was inoculated with 1 × 10^7^ CFU/mL of bacterial suspension adjusted to 0.5 McFarland. MIC was identified as the lowest concentration showing no visible turbidity, whereas MBC was defined as the lowest concentration producing no bacterial growth upon subculture. Measurements were performed for both *S. aureus* and *E. coli*.

## Ferrite characterization

The synthesized Cu_0.15_Zn_0.2_Ni_0.65_Fe_2_O_4_ ferrite, along with the reference single-cation ferrites CuFe_2_O_4_, NiFe_2_O_4_, and ZnFe_2_O_4_, was characterized using a combination of structural, optical, morphological, and magnetic techniques. X-ray diffraction (XRD) patterns of all samples were obtained with a MiniFlex 600 diffractometer (Rigaku, Japan) using Cu-Kα radiation (λ = 0.15406 nm) at 40 kV and 15 mA over 2θ = 10°–90° with a 0.02° step size and 2 s dwell time per step to confirm the crystalline phases. Fourier transform infrared (FTIR) spectra of all samples were recorded on a Bruker Alpha II spectrometer in the range 4000–400 cm^−1^ with 4 cm^−1^ resolution to identify characteristic vibrational bands. Optical properties of the Cu_0.15_Zn_0.2_Ni_0.65_Fe_2_O_4_ ferrite were investigated via UV–Vis. spectroscopy using an Edinburgh DS5 dual-beam spectrophotometer over 190–800 nm. K-Alpha X-ray Photoelectron Spectrometer XPS (Thermo Fisher Scientific - US) analysis should be performed to provide detailed information on the surface composition and oxidation states. Surface morphology was examined by scanning electron microscopy (SEM) with a JEOL JSM-700 instrument, capturing micrographs at × 500 and 1000 × magnifications to assess particle features and agglomeration. The SEM system is equipped with an energy-dispersive X-ray spectroscopy (EDX) detector, which was employed to determine the elemental composition of the synthesized ferrite. Transmission electron microscopy (TEM) using a JEOL JEM-2100 was employed to visualize the internal structure and determine particle size distributions, confirming nanoscale dimensions and uniformity. Finally, magnetic properties of the Cu_0.15_Zn_0.2_Ni_0.65_Fe_2_O_4_ ferrite were measured at room temperature using a vibrating sample magnetometer (VSM, Oxford OX8JTL, England) to determine saturation magnetization, coercivity, and remanence. Accurate quantitative elemental quantitative analysis of the various ferrite sample was performed using a flame atomic absorption spectrophotometer (AAS, SpectrAA220, Varian, Australia).

## Results and discussion

### XRD

**Fig. 3:**
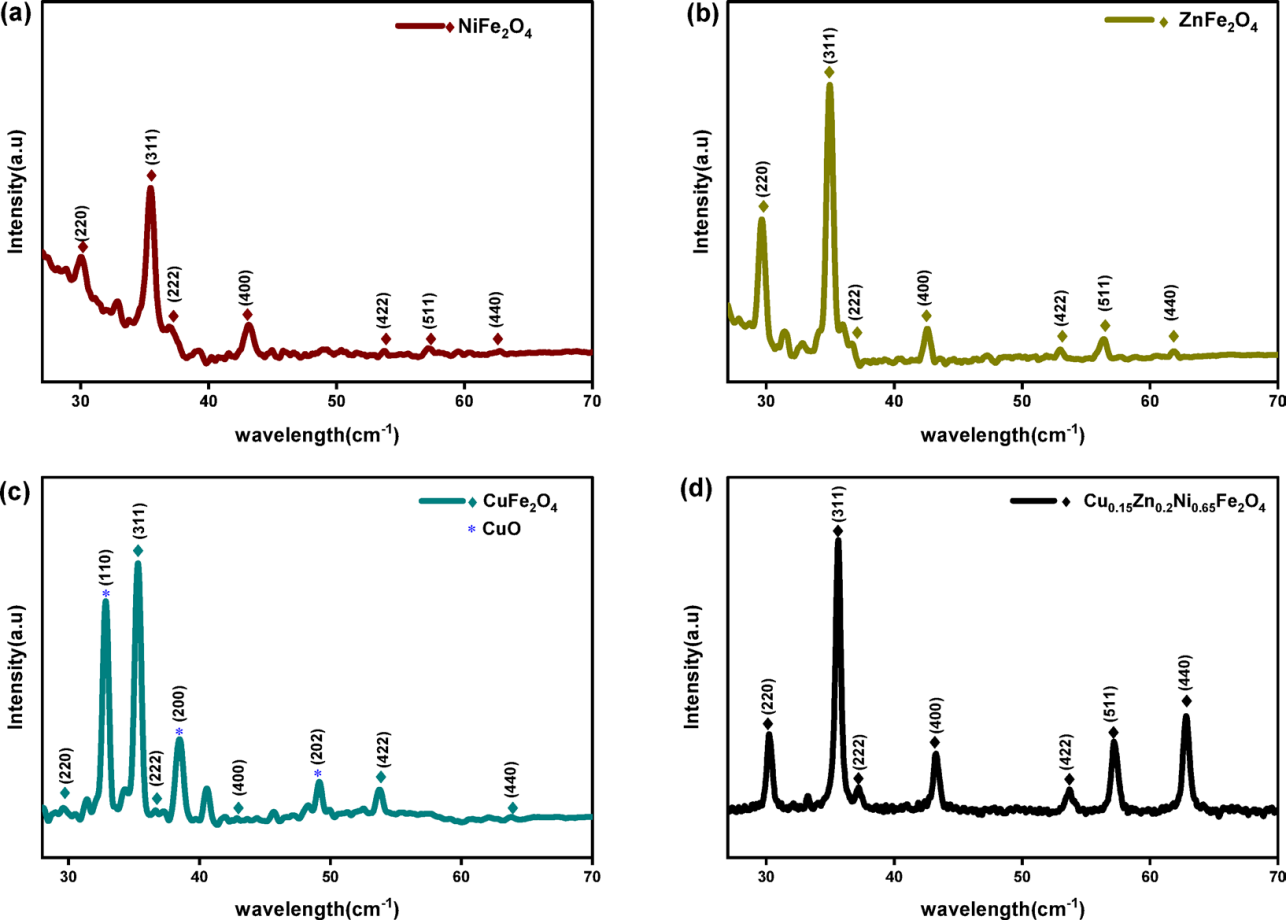
(**a**, **b**, **c**, and **d**). XRD of NiFe_2_O_4_, ZnFe_2_O_4_, CuFe_2_O_4_, and Cu_0.15_Zn_0.2_Ni_0.65_Fe_2_O_4_ nanoparticles, respectively.

The structures of the nanoferrites samples (NiFe_2_O_4_, ZnFe_2_O_4_, CuFe_2_O_4_, and Cu_0.15_Zn_0.2_Ni_0.65_Fe_2_O_4_) were analyzed using X-ray diffraction. The XRD pattern of the NiFe_2_O_4_ nanoparticles is presented in (Fig. 3a), the reflections at 30.5°, 36.04°, 37.7°, 43.8°, 54.3°, 57.9°, and 63.6°, corresponding to the (220), (311), (222), (400), (422), (511), and (440) planes, confirm the formation of a cubic spinel structure (PDF 74-1913; space group 227: Fd-3m)^50^. Similarly, the Zn Fe_2_O_4_ nanoparticles as shown in (Fig. 3b) exhibit characteristic peaks at 29.90°, 35.24°, 36.86°, 42.82°, 53.11°, 57.98°, and 62.16°, associated with the (220), (311), (222), (400), (422), (511), and (440) planes, confirming the formation of a cubic nickel spinel ferrite structure (PDF 81-0681; space group 227: Fd-3m)^51,52^.

For CuFe_2_O_4_ nanoparticles (Fig. 3c). The diffraction peaks observed at 2θ values of 30.06°, 35.41°, 37.04°, 43.03°, 53.39°, and 56.91°, corresponding to the (220), (311), (222), (400), (422), and (440) planes, respectively, confirm the formation of a cubic copper spinel ferrite phase (PDF 77-0427; space group 227: Fd-3m)^53–56^. Additional peaks indexed to the (110), (200), and (202) planes at 32.20°, 38.50°, and 49.20°, respectively, indicate the presence of CuO impurities (PDF 89-2530; space group 15: C2/c1)^56,57^.

XRD pattern of Cu_0.15_Zn_0.2_Ni_0.65_Fe_2_O_4_ is displayed in Fig. 3d. The pattern clearly reveals that the synthesized sample exhibits a single-phase cubic spinel structure, with no evidence of secondary phases or impurity peaks. Similarly, the XRD analysis confirms the formation of a pure spinel phase, with diffraction peaks at 30.20°, 35.57°,37.213°, 43.23°, 47.34°, 53.64°, 57.18°, and 62.80° corresponding to the (220), (311), (400), (222). (422), (511), (440), and (533) (DB Card Number 9012442, space group 227: Fd-3 m). The results obtained show excellent agreement with the standard reference data for the spinel phase listed under JCPDS card No. 74-2081^58–60^. The strong peak (311) reflection exhibits higher intensity, while the sharp and narrow diffraction peaks verify the high crystallinity and phase purity of the synthesized ferrite sample. The diffraction analysis confirms a cubic spinel structure assigned to the Fd_3_m space group^45^. For completeness, the full PDF reports of the XRD analysis including peaks indexing and phase matching has been included as Supplemental File 1.

Upon confirming the single-phase nature, further analysis was conducted on the structural parameters derived from the XRD data. The experimental lattice constant (a_exp_), crystallite size (D), theoretical lattice parameter, and cation distribution were determined.

#### Determination of lattice constant

For cubic crystal systems, the relationship between the interplanar spacing (d), the lattice constant (a) was determined using Cohen’s method^61^, (hkl) is the miller indices of the diffraction planes given by Eq. (1).

1 $${\mathrm{d}} = \frac{{\mathrm{a}}}{{\sqrt {{\mathrm{h}}^{{2}} + {\mathrm{K}}^{{2}} + {\mathrm{l}}^{{2}} } }}$$

(hkl) is the miller indices of the diffraction planes.

Bragg’s law describes the relationship between the interplanar spacing (d) and the diffraction angle (θ), and is expressed by Eq. (2)^44,61–63^:

2 $$\lambda = 2d sin\theta$$

Here, λ is the wavelength of Cu-Kα radiation, which is 1.5406 Å.

The lattice constant for the synthesized nanoferrites samples NiFe_2_O_4_, ZnFe_2_O_4_, CuFe_2_O_4_ is 8.2580, 8.4399, and 8.40, respectively (Table 1).

**Table 1:** Lattice constant of Cu_0.15_Zn_0.2_Ni_0.65_Fe_2_O_4_ nanoparticles.

λ (Å)	Bragg’s angle	Miller indices			Lattice constant (Å)	á (Å)
	2θ	h	k	l	a = $\left(d\sqrt{{h}^{2}+{K}^{2}+{l}^{2}}\right)$	
1.5406	30.18	2	2	0	8.3676	8.3642
	35.60	3	1	1	8.3571	
	43.25	4	0	0	8.3608	
	53.62	4	2	2	8.3668	
	57.14	5	1	1	8.3696	
	62.8	4	4	0	8.3635	

#### Crystallite size

The crystallite size of the synthesized samples was determined based on the broadening of the XRD diffraction peaks as shown in Table 2. The average crystallite size (D) was calculated using the Scherrer Eq. (3)^64^.

3 $${\mathrm{D}} = \frac{{{\mathrm{K}}\uplambda }}{{{\upbeta }\cos {\uptheta }}}$$

**Table 2:** indicates the crystallite size (D) of Cu_0.15_Zn_0.2_Ni_0.65_Fe_2_O_4_.

(2θ)	β (FWHM)	Crystalline size D (nm)	Average (nm)
30.185	0.4670	17.6191	15.8021
35.601	0.4770	17.4921	
43.25	0.5100	16.7564	
53.62	0.9700	9.1764	
57.14	0.5700	15.8698	
62.8	0.5200	17.8987	

For spinel ferrites, the shape factor (k) is typically taken as 0.9. The wavelength of the incident X-ray beam (λ) is 1.54 Å, corresponding to Cu Kα radiation. The term ‘β’ denotes the full width at half maximum (FWHM) of the most intense diffraction peak, and ‘θ’ refers to the Bragg angle associated with that peak. The crystallite sizes of the synthesized NiFe_2_O_4_, ZnFe_2_O_4_, and CuFe_2_O_4_ samples were found to be 18.17 nm, 16.03 nm, and 17.87 nm, respectively.

#### Cation distribution

The cation distribution within the spinel ferrite structure was analyzed based on XRD data, applying the Bertaut method^65,66^. This method enables the determination of how different cations are distributed between the tetrahedral (A) and octahedral (B) sites. According to Eq. (4), it is commonly known that Ni^2+^ ions predominantly occupy octahedral (B) sites. Additionally, Cu^2+^ ions exhibit a strong preference for octahedral sites. This trend aligns with the cation distribution model proposed by Shirsath et al.^40,67,68^. Whereas Zn^2+^ ions preferentially occupy tetrahedral sites due to their lower site preference energy in that coordination. The cation at A lattice is ferric Fe^3+^ and small traces of nickel ions where the other cations are present in B-site.

4 $$( Zn_{1 - t }^{2 + } {\text{ Fe}}_{{\mathrm{t}}}^{3 + } )\left[ {{\mathrm{Ni}}_{{2 - {\mathrm{x}} - {\mathrm{t}}}}^{2 + } {\mathrm{Cu}}_{{\mathrm{x}}}^{2 + } {\mathrm{Fe}}_{{\text{t }}}^{3 + } } \right]$$

where t and x are the concentration of ions at the A and B sites of Fe^3+^ and Cu^2+^.

The average ionic radii at sites A and B, denoted as r_A_ and r_B_ respectively by Eqs. (5), and  (6) and Equation were calculated using established formulas as described in previous literature^69,70^.

5 $${\boldsymbol{r}}_{{\boldsymbol{A}}} = \left( {{\text{ C}}_{{{\mathrm{Zn}}^{2 + } { }}}^{{\mathrm{A}}} } \right)\left( {r_{{{\mathrm{Zn}}^{2 + } }} { }} \right) + \left( {C_{{{\mathrm{Fe}}^{{3 + { }}} { }}}^{A} } \right)\left( {r_{{{\mathrm{Fe}}^{{3 + { }}} }} { }} \right)$$

6 $${\boldsymbol{r}}_{{\boldsymbol{B}}} { } = \left[ {\left( {{\text{ C}}_{{{\mathrm{Ni}}^{2 + } { }}}^{{\mathrm{B}}} } \right)\left( {r_{{{\mathrm{Ni}}^{2 + } }} { }} \right) + \left( {{ }C_{{{\mathrm{Cu}}^{2 + } { }}}^{B} } \right)\left( {r_{{{\mathrm{Cu}}^{2 + } }} { }} \right) + \left( {C_{{{\mathrm{Fe}}^{{3 + { }}} { }}}^{B} } \right)\left( {r_{{{\mathrm{Fe}}^{{3 + { }}} }} { }} \right){ }} \right]/2$$

where the ionic radii of the Zn^2+^, Cu^2+^, ${\mathrm{Ni}}^{2 + }$ and Fe^3+^ ions are denoted by $r_{{{\mathrm{Zn}}^{2 + } }}$, $r_{{{\mathrm{Cu}}^{2 + } }}$, $r_{{{\mathrm{Ni}}^{2 + } }}$ and $r_{{{\mathrm{Fe}}^{3 + } }}$, respectively. CA and CB indicate the ionic con centration at the A and B sites, from these values, the average ionic radii were determined to be rA equal 0.66 Å and *r*_*B*_ equal 0.663 Å.

The theoretical lattice constant (a_th_) can then be calculated accordingly based on the ionic radii and cation distribution as Eq. (7)^71,72^:

7 $${\mathbf{a}}_{{{\mathbf{th}}}} = \frac{8}{3\sqrt 3 }\left[ {\left( {{\mathrm{r}}_{{\mathrm{A}}} + {\mathrm{r}}_{{\mathrm{o}}} } \right) + \sqrt {3 } \left( {{\mathrm{r}}_{{\mathrm{B}}} + {\mathrm{r}}_{{\mathrm{o}}} } \right)} \right]$$

Here, a_th_ represents the theoretical lattice constant, r_o_ denotes the ionic radius of the oxygen ion (1.32 Å), while r_A_ and r_B_ correspond to the average ionic radius at the tetrahedral (A) and octahedral (B) sites, respectively. These radii are determined based on the specific distribution of cations occupying the A and B sublattices within the spinel structure. The calculated theoretical lattice constant (*a*_th_) is found to be approximately 8.336 Å, which is in close agreement with the experimentally determined lattice parameter.

The oxygen positional parameter (*u*), representing the distance between cations and oxygen anions can be calculated using the lattice constant (*a*), the ionic radius of the oxygen ion (*r*_o_ = 1.32 Å), and the average radius of cations in the octahedral sites (*r*_*B*_), (u) approximately equal 0.387 Å according to the Eq. (8)^73^:

8 $${\mathbf{r}}_{{\mathbf{B}}} = \left( {\frac{5}{8} - u} \right)(a{-}{\mathrm{r}}_{{\mathrm{o}}} )$$

As the length of Fe^3+^–O^2−^ bond at A and B sites slightly differ, and because cation substitution modifies these bond lengths through changes in ionic radii and local coordination, corresponding shifts in the tetrahedral (ν_1_) and octahedral (ν_2_). M–O stretching bands are expected and are confirmed by the FTIR analysis.

### FTIR

**Fig. 4:**
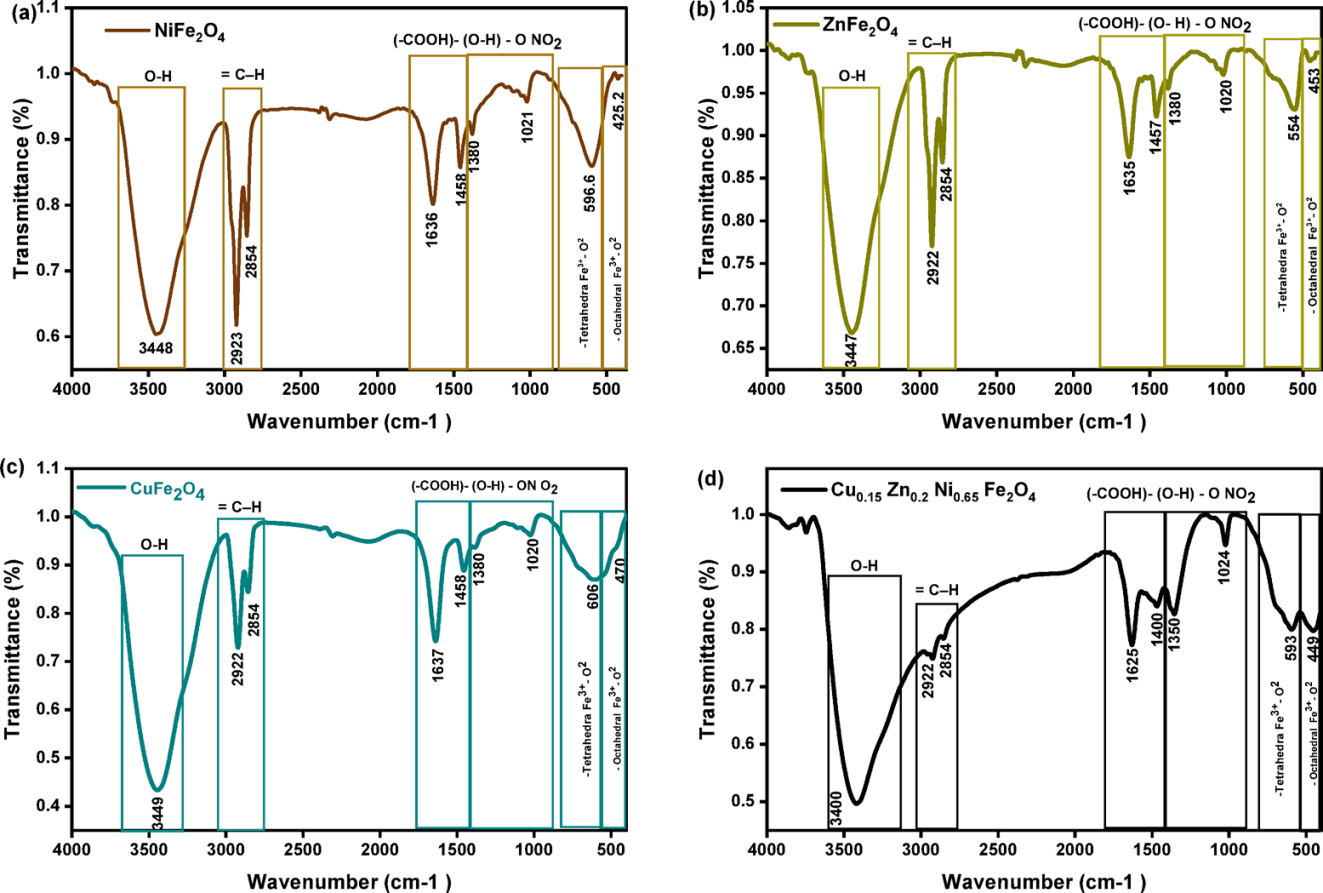
(**a**, **b**, **c**, and **d**). FTIR of NiFe_2_O_4_, ZnFe_2_O_4_, CuFe_2_O_4_, and Cu_0.15_Zn_0.2_Ni_0.65_Fe_2_O_4_ nanoparticles, respectively.

Fourier Transform Infrared transmittance spectra of the synthesized samples NiFe_2_O_4_, ZnFe_2_O_4_, CuFe_2_O_4_, and Cu_0.15_Zn_0.2_Ni_0.65_Fe_2_O_4_ nanoparticles are shown in (Fig. 4a–d), the range of spectra from 400 to 4000 cm^−1^. The spectra exhibit two prominent absorption bands, characteristic of spinel ferrite structures, corresponding to the vibrational modes of metal–oxygen (M–O) bonds, for NiFe_2_O_4_ (Fig. 4a), the absorption bands observed in as a strong band near 596.6 and a band near 425 cm^−1^, the prominent bands of ZnFe_2_O_4_ as shown in (Fig. 4b) appear at 554 and 453 cm^−1^, (Fig. 4c) presents CuFe_2_O_4_ bands at 606 and 470 cm^−1^, and Cu_0.15_Zn_0.2_Ni_0.65_Fe_2_O_4_ (Fig. 4d) sample exhibits bands around 593 and 449 cm^−1^, all of which are common features of spinel ferrite structures. The separation between the *ν*_1_ and *ν*_2_ bands is attributed to vibrations at the tetrahedral and octahedral sites^74^. These FTIR results confirm the formation of the spinel ferrite structure, which is typically identified by absorption features associated with Fe^3+^–O^2−^ bond vibrations in both tetrahedral and octahedral positions^70,75^.

Noted that, the *ν*_1_ and *ν*_1_ positions vary among the single-cation ferrites and show additional shifting in Cu_0.15_Zn_0.2_Ni_0.65_Fe_2_O_4_ sample. These shifts arise according to the difference of bond length of Fe^3+^–O^2−^ between tetrahedral and octahedral coordination, and substitution with positive ions of different ionic radii modifies the local Fe–O environment. Redistribution of Zn^2+^ to A-sites and Ni^2+^/Cu^2+^ to B-sites alters the bond force constants, resulting in measurable changes in both the frequency and relative intensity of the *ν*_1_ and *ν*_1_ bands. The FTIR spectra of all samples exhibit OH and N–O vibrational bands, indicating surface hydroxyl groups and nitrate-related residues, both of which are known to introduce localized states within the band structure and shift the absorption edge. The spectra display absorption bands in the range of 1020–1380 cm^−1^, corresponding to nitrate (NO_3_^−^) ions, and in the 1400–1637 cm^−1^ range, linked to carboxyl (COO^−^) groups. The peaks near 2927 and 2855 cm^−1^ are attributed to the antisymmetric and symmetric CH_2_ vibrations of carbon chains. A broad band around 3400–3450 cm^−1^ is attributed to hydroxyl (O–H) groups involved in hydrogen bonding. The specific vibrational modes identified from the FTIR analysis are summarized in Table 3.

**Table 3:** summarized the identified absorption bands and diffraction peaks, along with their corresponding assignments.

Wavelength	Modes and bonds	References
593–606 cm^−1^	Stretching vibrations of Fe^3+^–O^2−^ bonds in tetrahedral coordination sites	^76,77^
425–470 cm^−1^	Stretching vibrations of Fe^3+^–O^2−^ bonds in octahedral coordination sites	^76,77^
1020–1380 cm^−1^	Attributed to the vibrational mode of nitrate (NO_3_^−^) ions	^60,72^
1400–1637 cm^−1^	Absorption bands corresponding to carboxyl group (COO–)	^78,79^
2850–2930 cm^−1^	Antisymmetric and symmetric CH_2_ vibrations of carbon chains	^72,80,81^
3400–3450 cm^−1^	Assigned to the stretching mode of hydroxyl (OH) groups	^73^

### UV–Visible spectroscopy

**Fig. 5:**
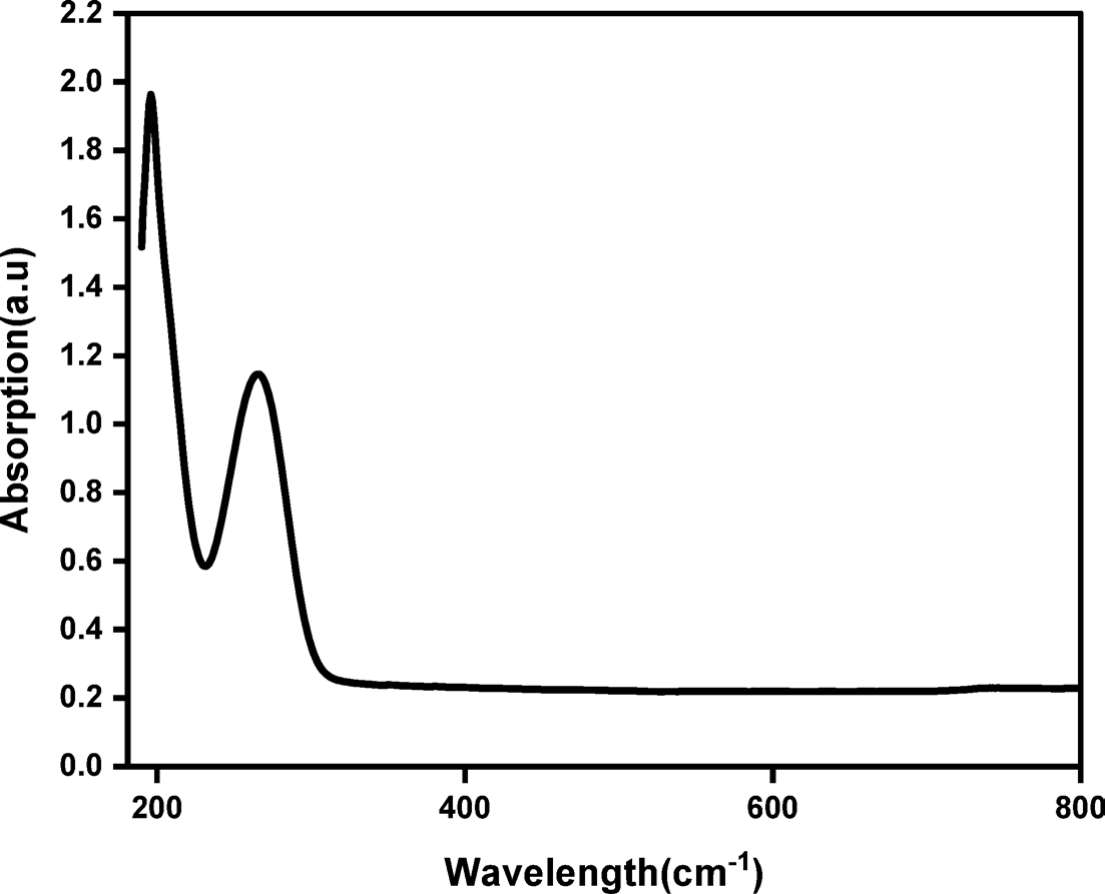
UV–visible absorbance spectra of Cu_0.15_Zn_0.2_Ni_0.65_Fe_2_O_4_ nanoparticles.

UV–Vis. spectroscopy was utilized to explore the optical properties of the synthesized Cu_0.15_Zn_0.2_Ni_0.65_Fe_2_O_4_ ferrite nanoparticles. The absorption spectra of the sample was measured at room temperature within the wavelength range of 200–800 nm, as shown in Fig. 5. The results indicate strong absorption with pronounced peaks appearing near 200 and 300 nm. Similar to other reported spinel ferrites^82–84^, these features are generally attributed to photo-induced electron excitations across the band gap, which attributed n-*π*^∗^ transitions. This absorption attributes to the intrinsic band gap absorption of an electron from valence band (VB) to the conduction band (CB)^85^.

**Fig. 6:**
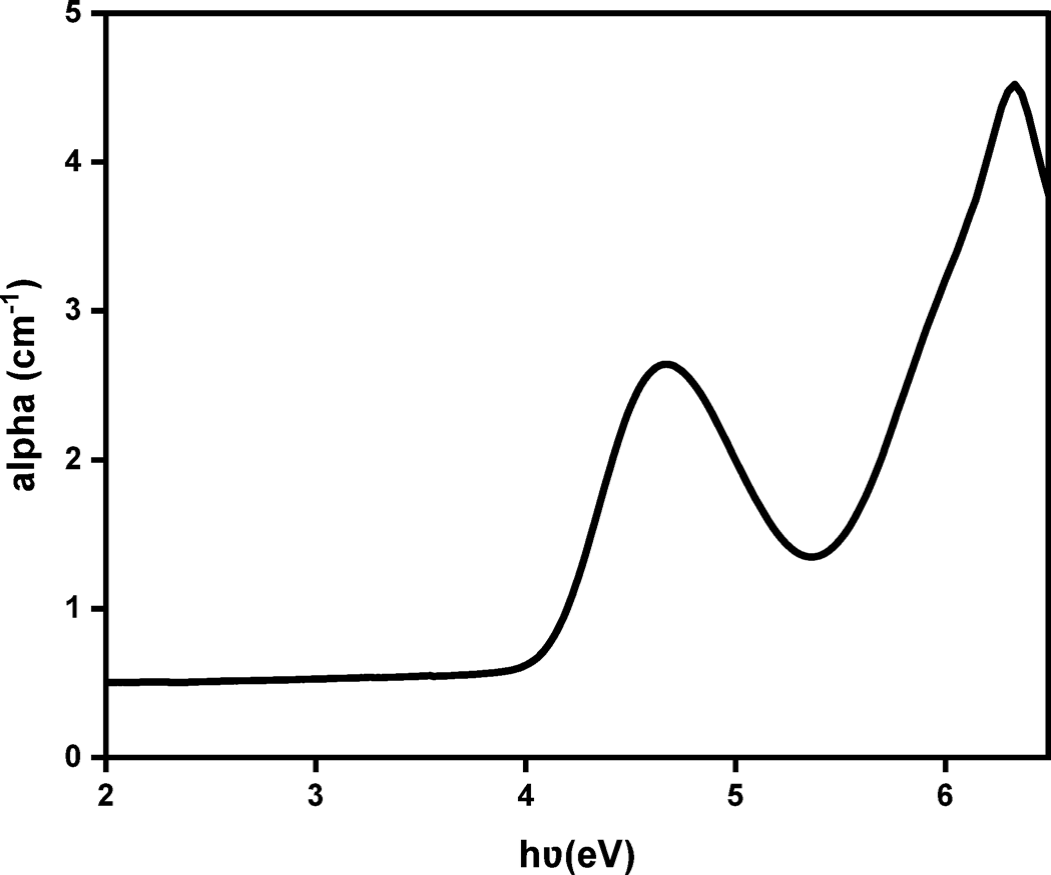
Energy-dependent absorption coefficient curve for Cu_0.15_Zn_0.2_Ni_0.65_Fe_2_O_4_ nanoparticles.

The absorption coefficient (α) was calculated based on absorbance measurements using the following Eq. (3)^86^ (Fig. 6):

9 $${{\boldsymbol{\upalpha}}} = \frac{{{\mathrm{absorbance}}}}{{\mathrm{d}}}$$

where d is the sample thickness, which was 1 cm due to the use of a quartz cuvette.

**Fig. 7:**
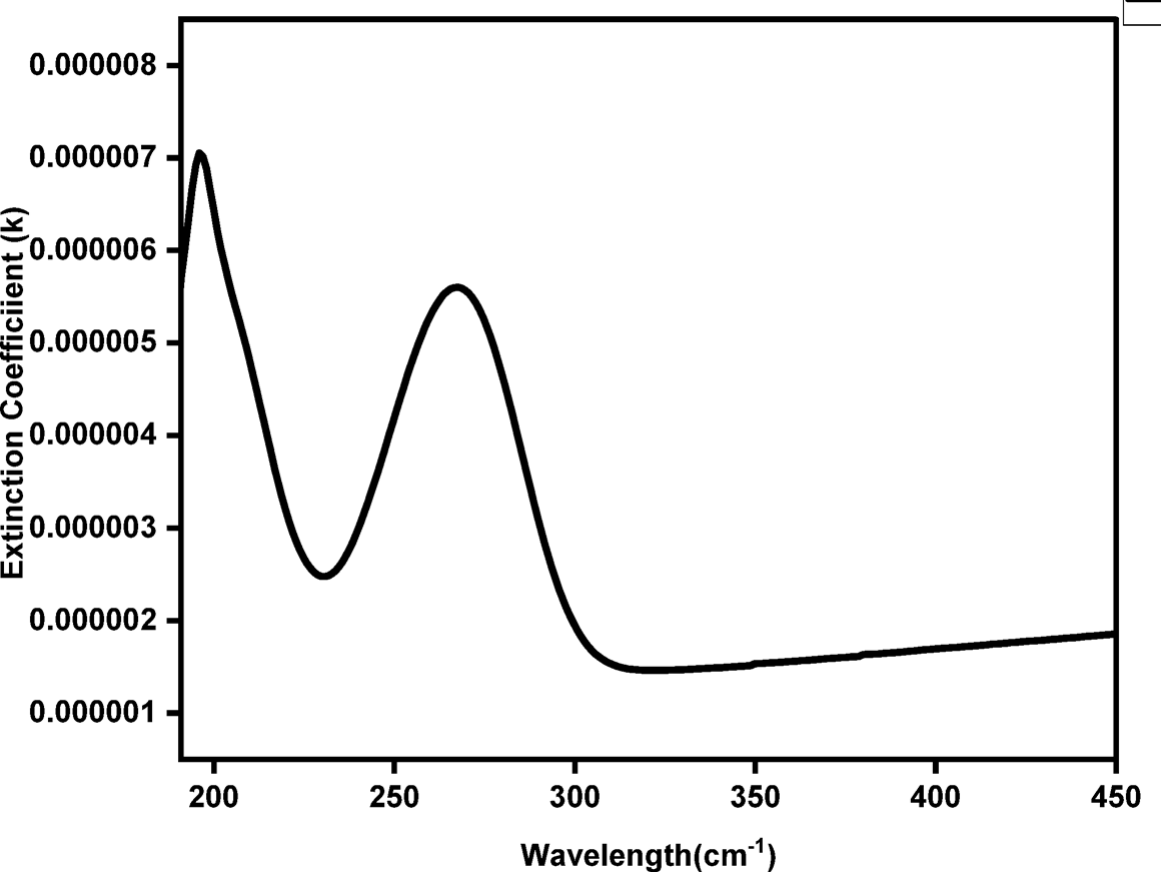
The extinction coefficient (k) vs wavelength for Cu_0.15_Zn_0.2_Ni_0.65_Fe_2_O_4_ nanoparticles.

The attenuation coefficient (*k*), often referred to as the extinction coefficient or absorption index, describes the degree to which the electric field amplitude diminishes as electromagnetic radiation propagates through a material as shown in Fig. 7. Its magnitude can be calculated using Eq. (4), as reported in Ref^87^:

10 $${\boldsymbol{k}} = \frac{\alpha \lambda }{{4\pi }}$$

where *α* is the absorption coefficient and *λ* is the wavelength of the incident light.

The optical band gap (Eg) of Cu_0.15_Zn_0.2_Ni_0.65_Fe_2_O_4_ was estimated using Tauc’s approach, which establishes a relationship between the absorption coefficient (α), the incident photon energy (hν), and the band gap energy. The Tauc relation is expressed in Eq. (11)^88,89^:

11 $$\left( {{\mathbf{\alpha} \textbf{h} \mathbf{\nu} }} \right)^{{1/n}} = {{\upalpha }}_{{\mathrm{o}}} \left( {{\mathrm{h}\upsilon } - {\mathrm{E}}_{{\mathrm{g}}} } \right)$$

In this model, h is Planck’s constant, ν is the photon frequency, and the exponent n depends on the type of electronic transition: *n* = 1/2 for direct allowed *n* = 2 for indirect allowed, *n* = 3/2 for direct forbidden, and *n* = 3 for indirect forbidden transitions. since both ZnFe_2_O_4_and NiFe_2_O_4_ are reported to exhibit direct band gap behavior^90,91^, the investigated ferrite system was also considered to follow a direct transition mechanism. Accordingly, the band gap was determined by extrapolating the straight line of the (αhν)^2^ = 0^92^.

**Fig. 8:**
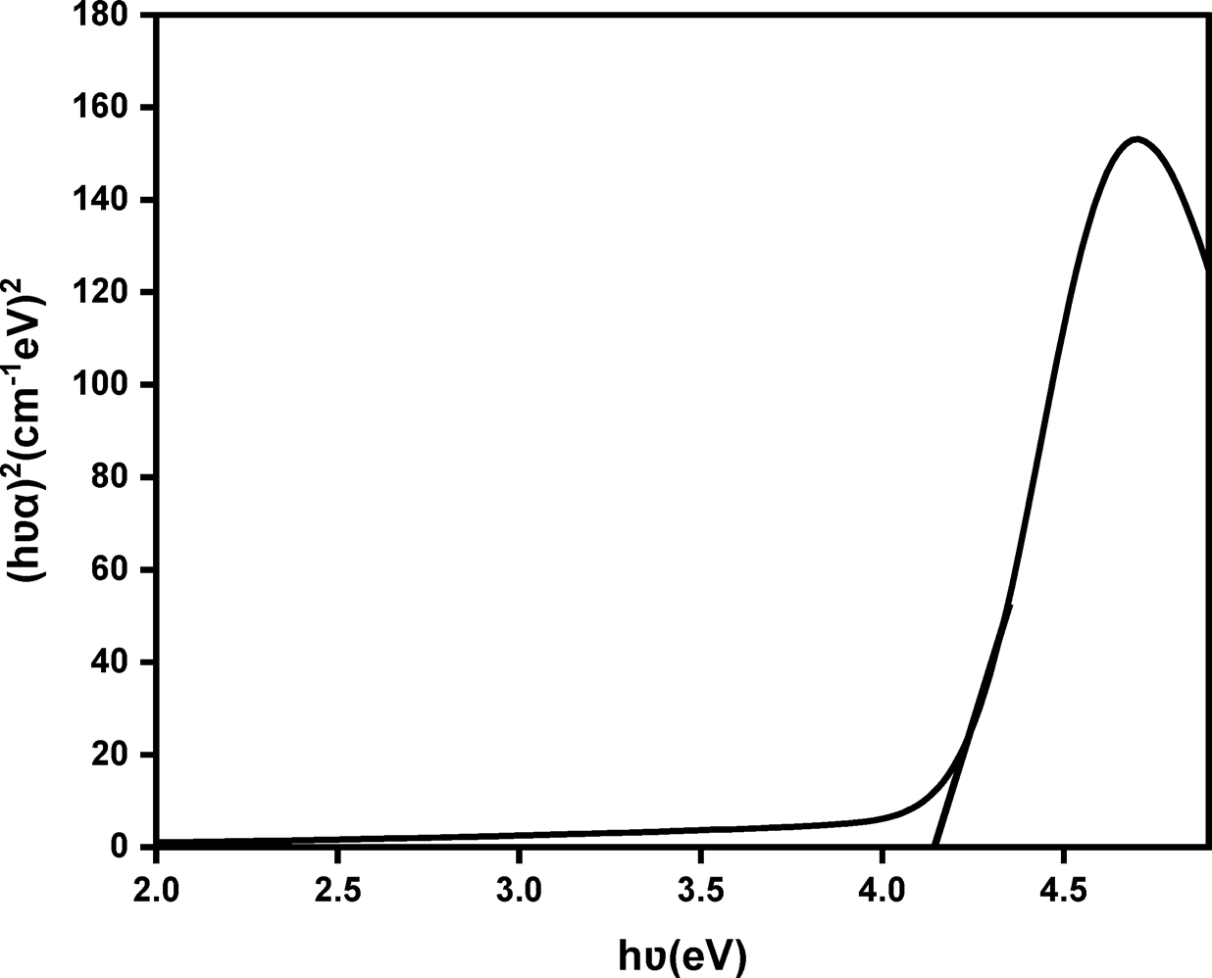
A plot of (αhν)^2^ versus hν of Cu_0.15_Zn_0.2_Ni_0.65_Fe_2_O_4_ nanoparticles.

In Fig. 8, the obtained value of Eg was approximately 4.1eV. Similar band-gap values (≈ 3.8–3.9 eV) have been observed in Cu-containing ferrites, where small crystallite size and quantum confinement contribute to a widening of the band gap^93^. Studies on Ni–Co ferrites have likewise shown that reducing particle size can shift ${\mathrm{E}}_{\mathrm{g}}$ toward higher energies due to the formation of discrete energy levels in the nanoscale regime^94,95^. In contrast, systems enriched with Zn such as Mg_1−x_Zn_x_ Fe_2_O_4_ and Mn_1−x_Zn_x_ Fe_2_O_4_, often exhibit further band-gap enlargement (≈ 4.0–5.0 eV)^96,97^, which has been linked to changes in lattice constant and modifications of the Fe–O bond environment caused by Zn substitution^92,98,99^. Reports on ZnFe_2_O_4_ nanoparticles similarly show band-gap values around 4 eV depending on particle size and thermal treatment^100^. The band gap obtained in the present study (~ 4.1 eV), together with the strong absorption feature near 306 nm, is therefore consistent with previously observed trends in Zn-rich and nanoscale ferrites. This behavior may arise from quantum-size effects and the introduction of defect-related energy states associated with Cu incorporation and oxygen-related groups, as indicated by the FTIR spectra.

### XPS analysis

**Fig. 9:**
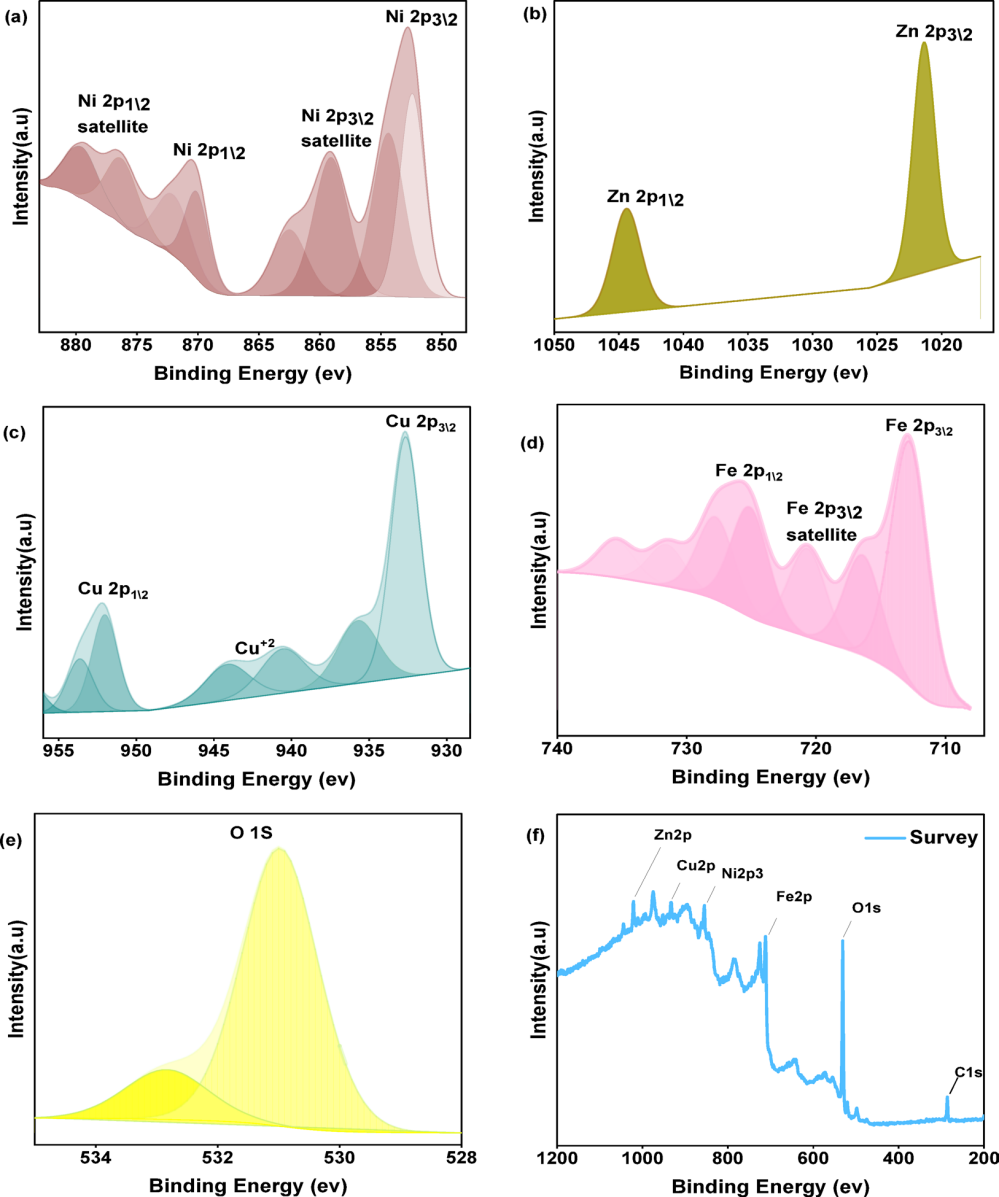
(**a**–**f**). XPS spectra of Cu_0.15_Zn_0.2_Ni_0.65_Fe_2_O_4_ nanoparticles (**a**) Ni 2*p*_,_ (**b**) Zn 2*p* (**c**) Cu 2*p* and (**d**) Fe 2*p*, (**e**) O 1*s*, and (**f**) Survey Plot, respectively.

To further characterize the synthesized sample, its chemical composition and the electronic states of the constituent elements in Cu_0.15_Zn_0.2_Ni_0.65_Fe_2_O_4_ were examined using XPS analysis. Figure 9 displays the XPS spectra of Cu_0.15_Zn_0.2_Ni_0.65_Fe_2_O_4_, showing the expected signals from Zn, Cu, Ni, Fe, and O. The Ni 2*p* spectrum (Fig. 9a) exhibits four well-defined peaks at 852.7 eV, 860.0 eV, 872.6 eV, and 880.4 eV, corresponding to the Ni 2*p*_3\2_ and Ni 2*p*_1\2_ states, consistent with earlier reports^43,101^. The Zn 2*p* spectrum (Fig. 9b) shows two major peaks at 1021.4 eV (Zn 2*p*_3/2_) and 1044.5 eV (Zn 2*p*_1_/_2_), confirming the presence of Zn^2+^ ions in the lattice^102,103^. The Cu 2*p* spectra (Fig. 9c) display characteristic Cu features, with the Cu 2*p*_3/2_ peak at 932.7 eV accompanied by shake-up satellite peaks at approximately 940 eV, indicative of the 3*d*^9^ configuration of Cu^2+^ ions, and the Cu 2*p*_1_/_2_ peak at 952.1 eV. The Fe 2*p* spectrum (Fig. 9d) presents three main peaks at 712 eV, 720.5 eV, and 725.8 eV, assigned to Fe 2*p*_3/2_ (Fe^2+^), a satellite feature associated with Fe^3+^, and Fe 2*p*_1_/_2_, respectively^43,104^. The high-resolution O 1*s* spectrum (Fig. 9e) shows a peak at 530.8 eV corresponding to lattice oxygen, along with a peak at 532.8 eV attributed to oxygen vacancy-related species^66,103^. Overall, the observed oxidation states and valence distributions (Fig. 9a–e) agree well with previously reported values for similar ferrite systems^41^.

### SEM and EDX analysis

**Fig. 10:**
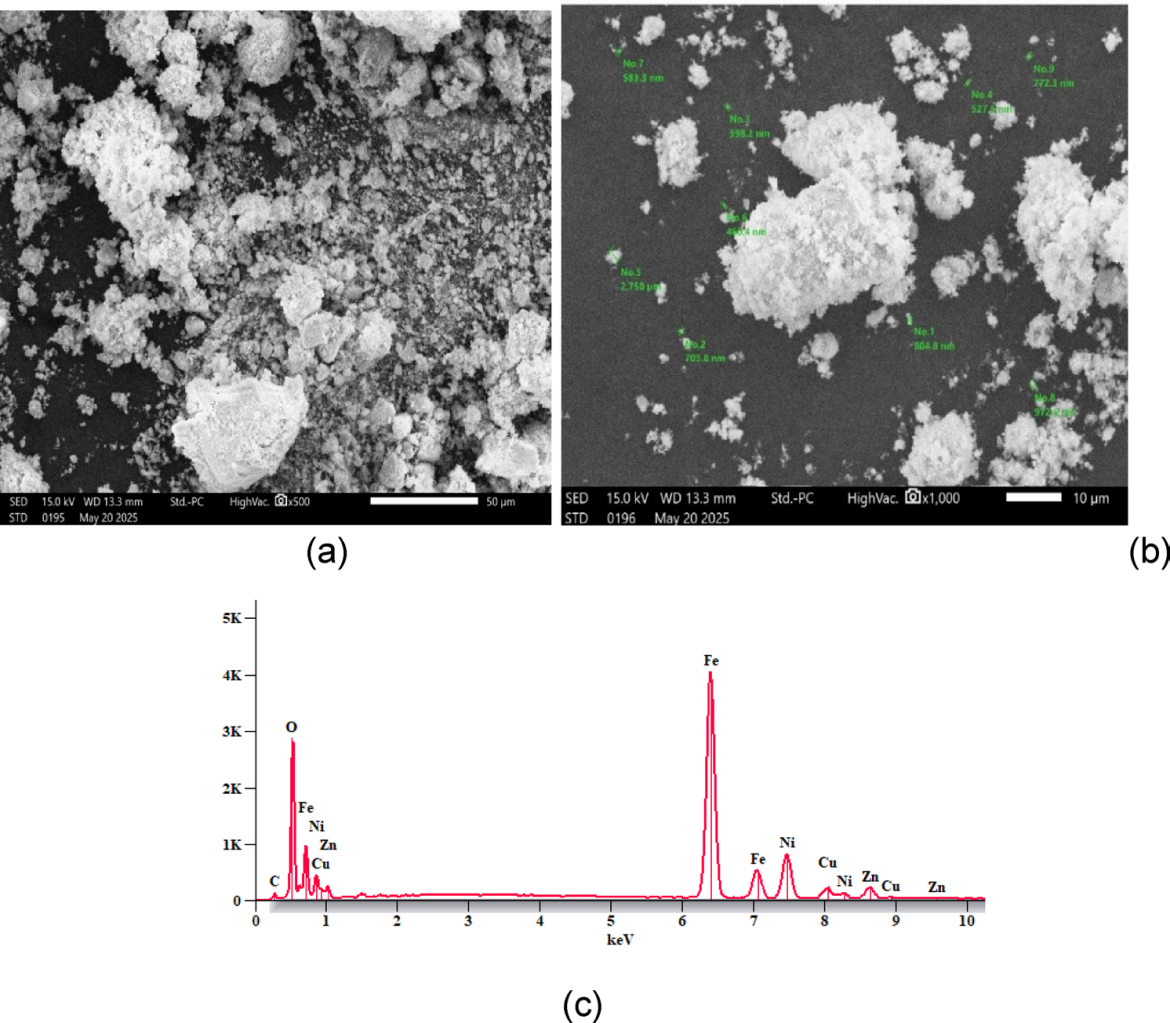
(**a**) SEM micrograph of Cu_0.15_Zn_0.2_Ni_0.65_Fe_2_O_4_ nanoparticles, (**b**) particle size distribution, and (**c**) EDX pattern of Cu_0.15_Zn_0.2_Ni_0.65_Fe_2_O_4_ nanoparticles, respectively.

SEM was employed to investigate the surface features of the synthesized Cu_0.15_Zn_0.2_Ni_0.65_ Fe_2_O_4_ nanoparticles as shown in (Fig. 10a,b). The micrographs reveal that the particles possess a predominantly spherical morphology with uniform spatial distribution. The nanoparticles exhibit a degree of agglomeration, which is a typical characteristic of magnetic nanomaterials and is primarily attributed to dipole–dipole magnetic interactions among the particles^69,74^. The grain size obtained from SEM has higher value than the corresponding values obtained from TEM and XRD, which indicate that every grain is formed composed of multiple crystallites, whereas the x-ray line broadening analysis yields only single crystallite size^74^. The grain size distribution determined from SEM images indicates an average approximately 608.4 nm, which is in good agreement with values reported in earlier studies^42,105–107^.

The stoichiometric analysis of the powder samples was carried out using EDX. The EDX spectra of the representative samples at 30 keV are shown in (Fig. 10c). Table 4 presents the elemental composition of the sample. The spectra indicated the presence of key elements such as Ni, Cu, Zn, Fe, C, and O, with no detected impurities. The carbon (C) observed in the spectra is a result of the carbon coating applied for SEM imaging before the EDX analysis.

**Table 4:** Presents the elemental composition of the sample.

C (Wt%)	O (Wt%)	Fe (Wt%)	Ni (Wt%)	Cu (Wt%)	Zn (Wt%)
1.4	20.9	54.9	12.9	4.3	5.7
2.2	25.6	49.3	13.6	4.2	5.0
11.1	7.4	58.7	13.6	3.6	5.5
0.9	17.6	57.1	13.9	4.1	6.4
1.6	21.4	52.1	14.6	4.5	5.8

### Atomic absorption spectroscopy analysis

To quantify the elemental content of Cu, Zn, and Ni in the synthesized Cu_0.15_Zn_0.2_Ni_0.65_Fe_2_O_4_ ferrite, Atomic Absorption Spectroscopy (AAS) was performed following complete dissolution of a known mass of the powder in dimethyl sulfoxide (DMSO). The clear solution was analyzed using a flame AAS instrument (SpectrAA220, Varian, Australia). Measurements were conducted using hollow-cathode lamps at the analytical wavelengths of Cu (222.6 nm), Zn (213.9 nm), and Ni (323.0 nm). Calibration standards were prepared for each ion, and quantification was carried out based on the corresponding absorbance–concentration calibration plots.

To support the discussion of ion-related antibacterial activity, the elemental content of Cu, Zn, and Ni in the synthesized ferrite was quantified. The measured concentrations were Zn (4.056 wt%), Cu (4.016 wt%), and Ni (1.767 wt%). These average values correspond to the total elemental content released after complete dissolution of the ferrite sample and confirm the presence of all relevant cations in measurable quantities. Although these measurements do not represent time-dependent ion-release kinetics, they demonstrate the availability of Cu^2+^, Zn^2+^, and Ni^2+^ ions in the material, supporting their proposed contribution to the antibacterial mechanism.

### TEM

**Fig. 11:**
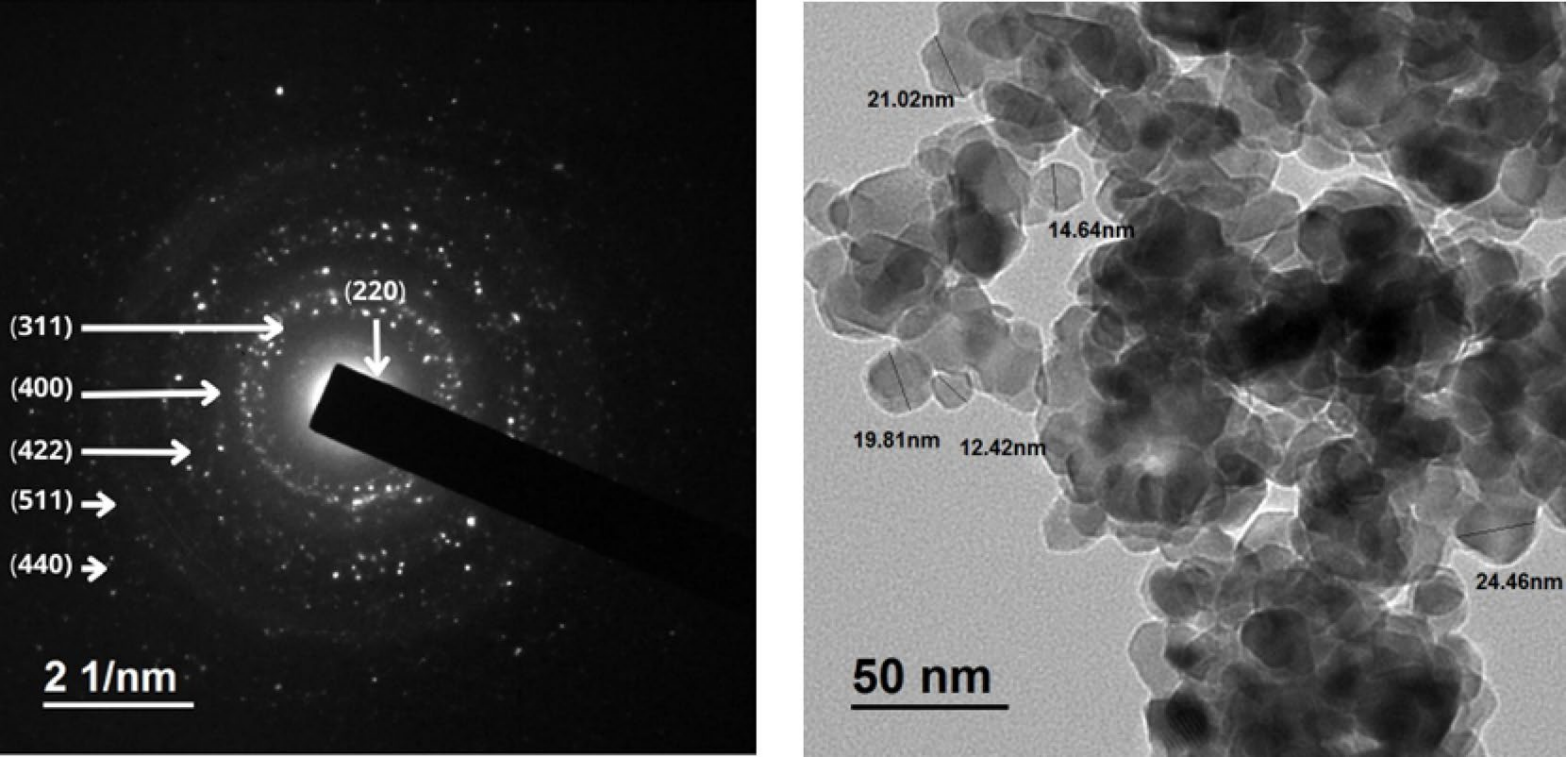
SAED Pattern and TEM image of Cu_0.15_Zn_0.2_Ni_0.65_Fe_2_O_4_ nanoparticles, respectively.

Figure 11 was employed to examine the morphology and dispersion behavior of the synthesized Cu_0.15_Zn_0.2_Ni_0.65_Fe_2_O_4_ nanoparticles. The structural characteristics inferred from XRD analysis were further validated through TEM observations. Figure presents TEM images revealing a nanocrystalline structure with evident agglomeration, likely caused by magnetic interactions among the nanoparticles. The crystallites exhibit a spherical morphology, and the particle sizes correspond well with those calculated from XRD.

The selected area electron diffraction (SAED) pattern reveals well-defined concentric rings (Fig. 11), which confirm the crystalline nature of the synthesized ferrite nanoparticles^108,109^. This diffraction rings correspond directly to the main reflections observed in the XRD patterns, indicating strong agreement between the electron and X-ray-based structural analyses. Additionally, the crystal size distribution histogram shows an average crystal size of about 22 nm, which is larger than the crystallite size calculated by Scherer’s equation^70^. The observed agglomeration could be due to magnetic interactions between nanoparticles^110^.

### VSM

**Fig. 12:**
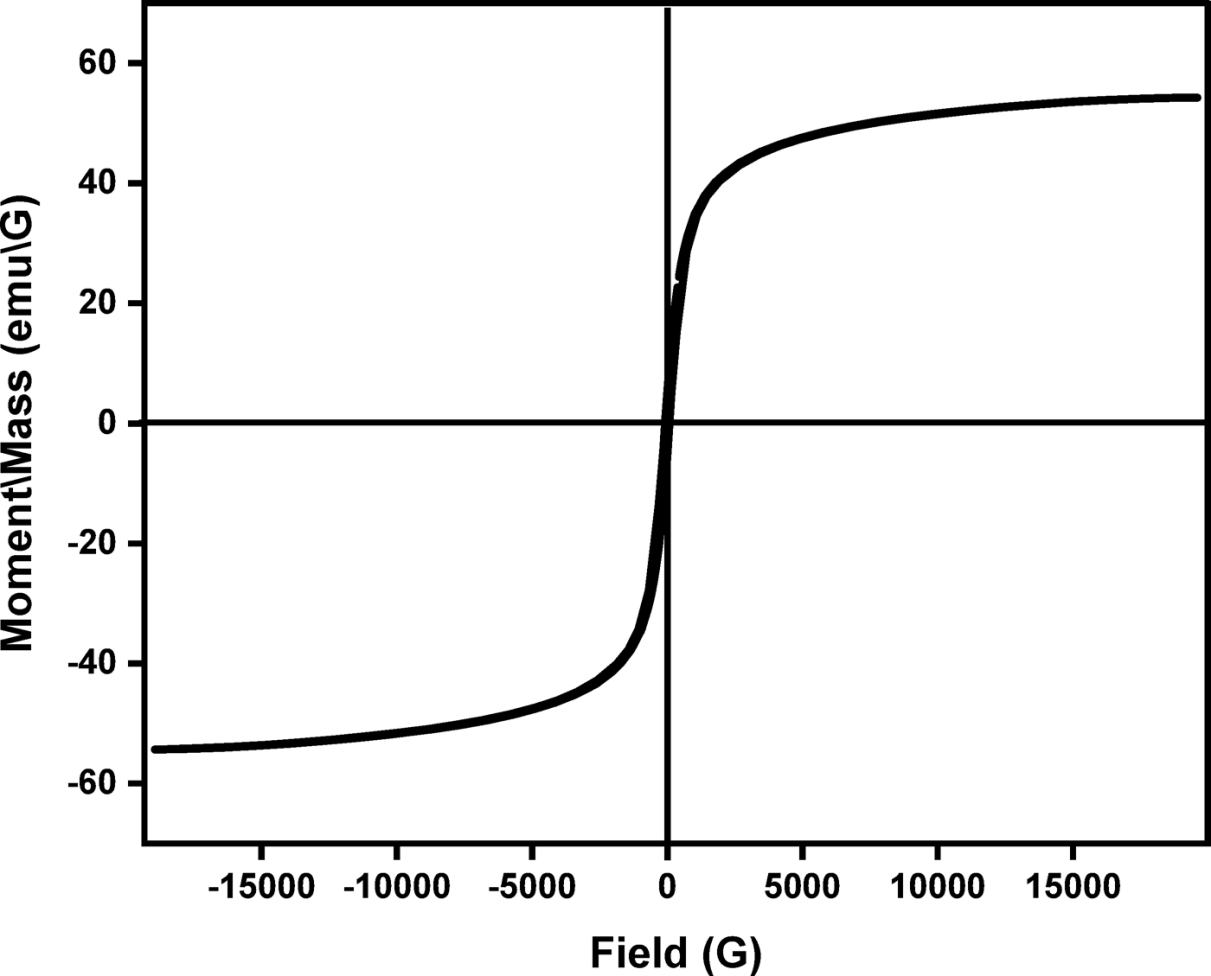
The (M − H) hysteresis loop of Cu_0.15_Zn_0.2_Ni_0.65_Fe_2_O_4_ nanoparticles.

The magnetic characteristics of Cu_0.15_Zn_0.2_Ni_0.65_Fe_2_O_4_ nanoparticles were evaluated using hysteresis loop measurements, in which magnetization was plotted as a function of the applied magnetic field Fig. 12. The loop reveals a relatively low coercive field (Hc ≈ 80.717 G), confirming that the prepared ferrite belongs to the class of soft magnetic materials. The magnetization response further confirms the ferrimagnetic behavior of the Cu_0.15_Zn_0.2_Ni_0.65_Fe_2_O_4_ nanoparticles. From the hysteresis loop, the extracted magnetic parameters are saturation magnetization (M_s_ ≈ 54.294 emu/g), remanent magnetization (M_r_ ≈ 5.247 emu/g), coercivity (H_c_ ≈ 80.717 G).

The experimental magnetic moment (μ_exp_) was calculated using the Eq. (12)^111^:

12 $${{\boldsymbol{\upmu}}}_{{{\mathbf{exp}}}} = \frac{{{\mathrm{M*M}}_{{\mathrm{s}}} }}{5585}$$

where M represents the molecular weight of the ferrite composition and M_s_ is the saturation magnetization in emu/g, the calculated μ_exp_ value was found to be 3.52 μ_B_.

The theoretical magnetic moment (μ_th_), obtained using Néel’s two-sublattice model^112^, is given by Eq. (13):

13 $${{\boldsymbol{\upmu}}}_{{{\mathbf{th}}}} =\upmu _{{\mathrm{B}}} {\mathrm{cos}}\uptheta _{{\mathrm{Y}}} - \mu_{{\mathrm{A}}}$$

where μ_B_ and μ_A_ ​ are the magnetic contributions of octahedral (B) and tetrahedral (A) sites, respectively, and γ is the canting angle of the B-site spins. The calculated μ_th_ value and γ, and all of other values summarized in Table 5.

**Table 5:** Present the magnetic parameters of Cu_0.15_Zn_0.2_Ni_0.65_Fe_2_O_4_ nanoparticles.

M_s_ (emu/g)	M_r_ (emu/g)	H_c_ (G)	µ_th_ (µ_B_)	µ_exp_ (µ_B_)	μ_A_ (µ_B_)	μ_B_ (µ_B_)	θ_Y_ (°)
54.294	5.247	80.717	3.223	3.52	3.912	7.135	53.8

### Antibacterial activity

**Fig. 13:**
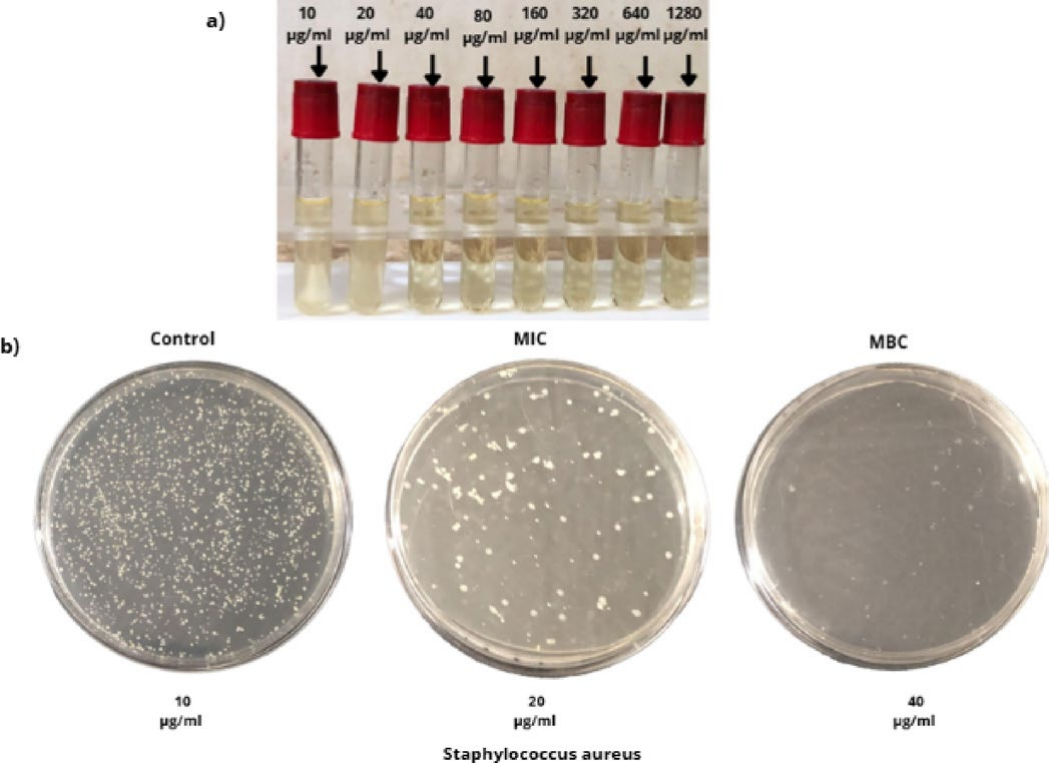
(**a**) Presents the serial dilution of Cu_0.15_Zn_0.2_Ni_0.65_Fe_2_O_4_ concentration from 10 to 1280 μg/ml. (**b**) MIC and MBC of Cu_0.15_Zn_0.2_Ni_0.65_Fe_2_O_4_ against *Staphylococcus aureus*.

**Fig. 14:**
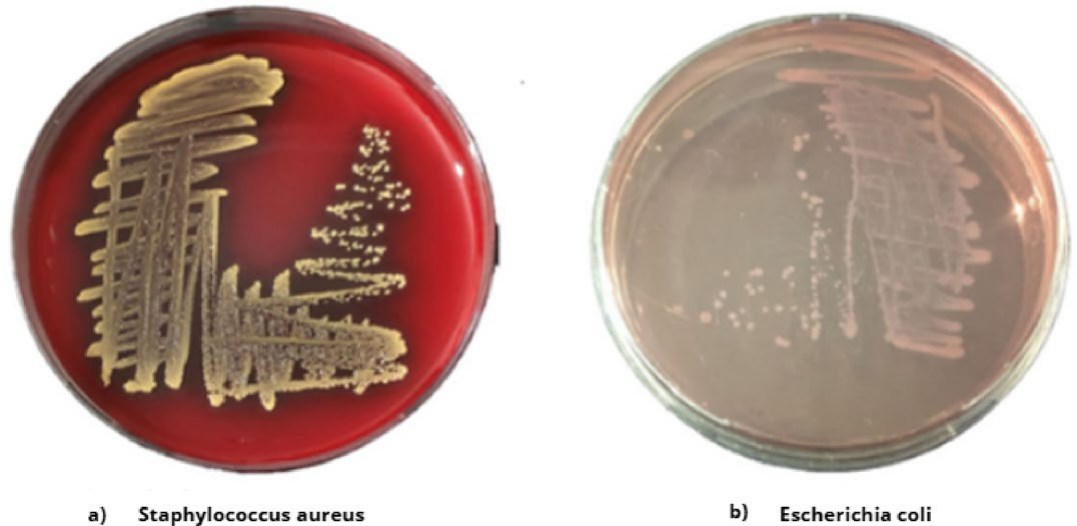
(**a**) Golden yellow color of *Staphylococcus aureus* colony on blood agar petri dish. (**b**) *Escherichia coli* on MacConkey agar petri dish.

For assessing the antibacterial efficacy of the Cu_0.15_Zn_0.2_Ni_0.65_Fe_2_O_4_ nanoparticles, we examined the antibacterial performance of NiFe_2_O_4_, ZnFe_2_O_4_, and CuFe_2_O_4_ comparable to Cu_0.15_Zn_0.2_Ni_0.65_Fe_2_O_4_ nanoparticles. Also, we observed the effect upon using different concentrations of Cu_0.15_Zn_0.2_Ni_0.65_Fe_2_O_4_ against *S. aureus* coli (gram-positive) and (gram-negative) *E. coli*. As shown in (Fig. 14a, and b) the bacterial strains colors and biochemical reactions of *S. aureus* (golden yellow and beta hemolytic strains) and *E. coli* (pink and non-lactose fermentation) on blood agar and MacConkey agar petri dish, respectively^113,114^. (Fig. 13a) presents the serial dilution of Cu_0.15_Zn_0.2_Ni_0.65_Fe_2_O_4_ nanoparticles concentration from 10 to 1280 μg/ml with adjusted bacterial suspension concentration at 1 × 10^7^ CFU/ml. We also determined MIC and the MBC for nanoparticles; Table 6 presents the impact of different concentrations upon *S. aureus* and *E. coli* (Fig. 14).

**Table 6:** Presents the growth of bacterial strains on nutrient broth.

Growth of bacterial strains on nutrient broth								
Cu_0.15_Zn_0.2_Ni_0.65_Fe_2_O_4_ nanoparticle concentration	10 μg/ml	20 μg/ml	40 μg/ml	80 μg/ml	160 μg/ml	320 μg/ml	640 μg/ml	1280 μg/ml
*Escherichia coli*	+	+	−	−	−	−	−	−
*Staphylococcus aureus*	+	−	−	−	−	−	−	−

After inoculation bacterial suspension from turbidity and clear tube on nutrient agar media and incubated for appropriate temperature and time, we obtained the MIC for nanoparticles against *E. coli* at concentration 40 μg/ml and the MBC at concentration 80 μg/ml, while MIC for nanoparticles against *S. aureus* at concentration 20 μg/ml and the MBC at concentration 40 μg/ml as shown in Fig. 13b.

**Fig. 15:**
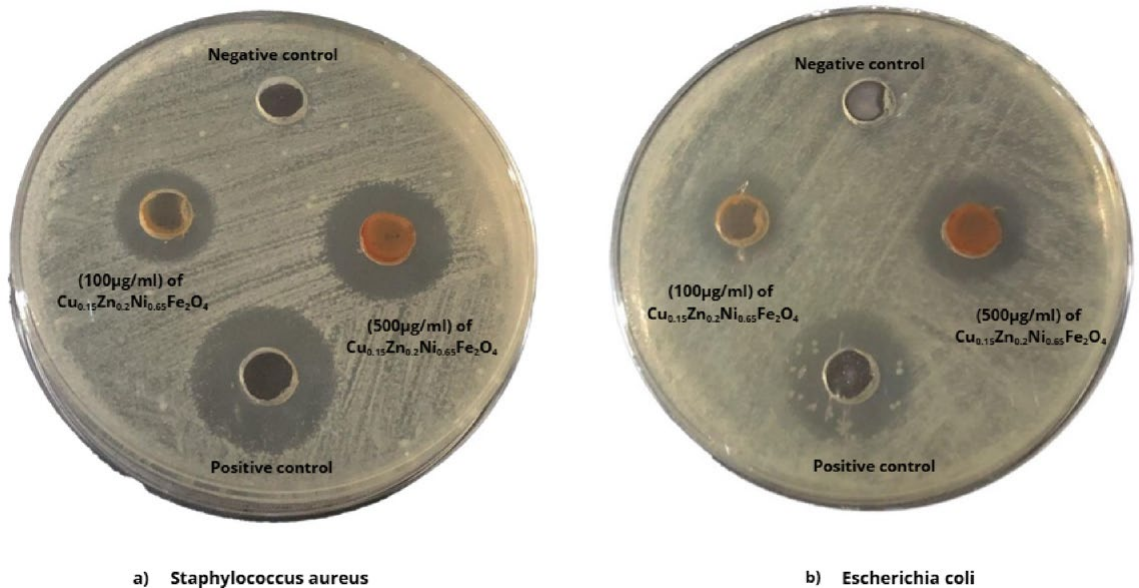
Zone of inhibition around wells of Cu_0.15_Zn_0.2_Ni_0.65_Fe_2_O_4_ (100, 500 μg/ml), gentamycin (positive control) and DMSO (Negative control) against (**a**) *Staphylococcus aureus* and (**b**) *Escherichia coli* bacterial strain, respectively.

In the analysis test of the inhibition zone, the highest in the sensitivity of the bacterial strain to the anti-bacterial material, the largest in the diameter of the bacterial inhibition zone. (Fig. 15a,b) represent the bacterial activity of Cu_0.15_Zn_0.2_Ni_0.65_Fe_2_O_4_ against *S. aureus* strain and *E. coli*, respectively. Also, the results are summarized in Table 7. Our results showed that DMSO (Negative control) did not inhibit the growth of the bacteria, whereas gentamycin (positive control) exhibited a relatively larger value than the zone of inhibition for Cu_0.15_Zn_0.2_Ni_0.65_Fe_2_O_4_ nanoparticle. Cu_0.15_Zn_0.2_Ni_0.65_Fe_2_O_4_ nanoparticle showed clear growth inhibitory effects against both *S. aureus* strain and *E. coli* in the experimental concentration range (100–500 µg/mL). The maximum inhibition zone was obtained against the *S. aureus* (20 ± 1.50 mm) and *E. coli* (17 ± 1.50 mm) at a concentration of 500 µg/mL.

**Table 7:** Antibacterial activity of Cu_0.15_Zn_0.2_Ni_0.65_Fe_2_O_4_ nano-ferrite against pathogenic bacteria.

Bacteria	Inhibition zone diameter (mm)			
	Cu_0.15_Zn_0.2_Ni_0.65_Fe_2_O_4_ (100μg/ml)	Cu_0.15_Zn_0.2_Ni_0.65_Fe_2_O_4_ (500μg/ml)	Positive control (Gentamycin)	Negative control (DEMSO)
*Staphylococcus aureus*	16 ± 1.0	20 ± 1.50	22 ± 1.0	Zero
*Escherichia coli*	13 ± 0.50	17 ± 0.50	20 ± 1.0	Zero

Observed that gram-negative bacteria cell wall is resistant than gram-positive bacteria cell wall. Gram-positive *S. aureus* bacteria at the concentration of 500 μg/ml obtained an inhibition zone equal to 20 mm and MIC at 40 μg/ml. On the other hand, gram-negative *E. coli* bacteria equal to17 mm and MIC equal 80 μg/ml*.* The difference in inhibition zones can be attributed to variations in bacterial cell–wall architecture. Gram-positive bacteria possess a thick peptidoglycan layer enriched with teichoic acids, which facilitates metal-ion binding and increases nanoparticle adhesion. In contrast, Gram-negative bacteria have an outer membrane containing lipopolysaccharide (LPS), which acts as a permeability barrier and reduces nanoparticle penetration. Accordingly, *S. aureus* exhibited larger inhibition zones and lower MIC/MBC values compared to *E. coli*, indicating higher susceptibility.

**Fig. 16:**
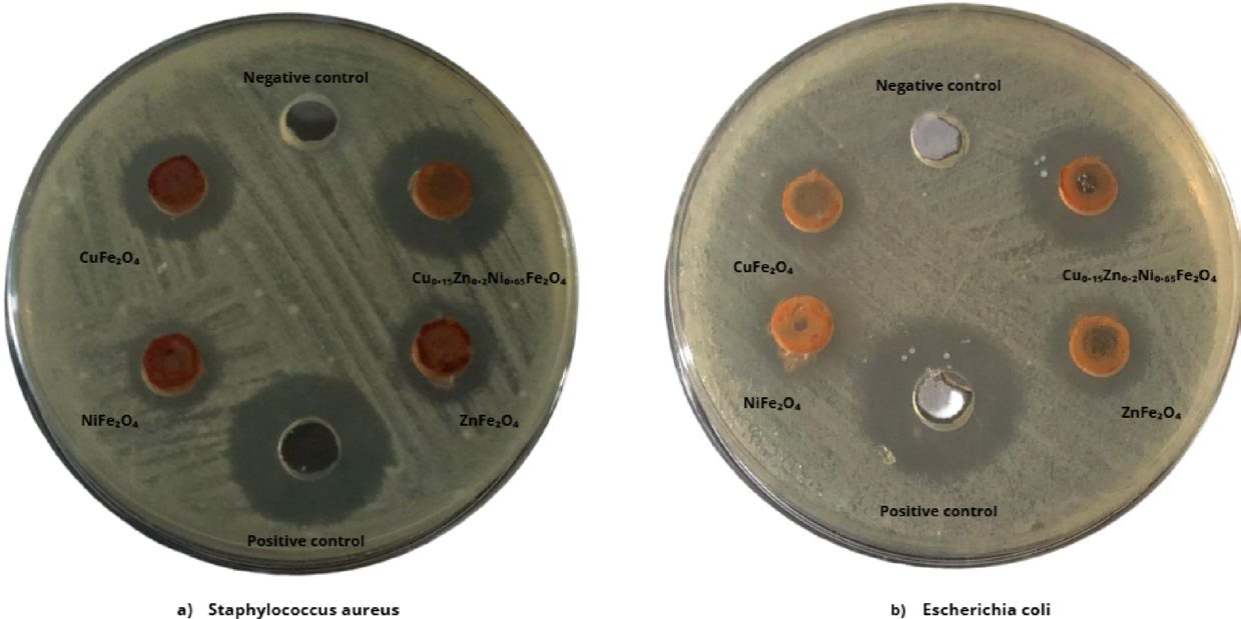
Comparison in Zone of inhibition around wells between CuFe_2_O_4,_ NiFe_2_O_4_, ZnFe_2_O_4_, and Cu_0.15_Zn_0.2_Ni_0.65_Fe_2_O_4_, gentamycin (positive control) and DMSO (Negative control) against (**a**) *Staphylococcus aureus* and (**b**) Escherichia coli bacterial strain, respectively.

To enable the comparison, all synthesized nano-ferrites (NiFe_2_O_4_, ZnFe_2_O_4_, CuFe_2_O_4_ and Cu_0.15_Zn_0.2_Ni_0.65_Fe_2_O_4_ nanoparticles) are poured as detailed in the assay in two MHA petri dishes against *S. aureus* and *E. coli* as depicted in (Fig. 16a,b) and summarized in Table 8.

**Table 8:** Antibacterial activity of NiFe_2_O_4_, ZnFe_2_O_4_, CuFe_2_O_4_ and Cu_0.15_Zn_0.2_Ni_0.65_Fe_2_O_4_ nanoparticles against pathogenic bacteria.

Inhibition zone diameter (mm)						
Nanoparticles concentration	NiFe_2_O_4_ (500 μg/ml)	ZnFe_2_O_4_ (500 μg/ml)	CuFe_2_O_4_ (500 μg/ml)	Cu_0.15_Zn_0.2_Ni_0.65_Fe_2_O_4_ (500 μg/ml)	Positive control (Gentamycin)	Negative control (DMSO)
*Staphylococcus aureus*	13 ± 0.50	14 ± 0.50	16 ± 1.0	20 ± 1.50	22 ± 1.0	Zero
*Escherichia coli*	12 ± 0.50	13 ± 0.75	14 ± 1.0	17 ± 1.50	21 ± 1.0	Zero

The inhibition zone is considered a marker of antibacterial effectiveness of all nano-ferrites. It’s obvious the superiority of the ternary structure Cu_0.15_ Zn_0.2_Ni_0.65_Fe_2_O_4_ that exhibited larger inhibition zone comparable to other single components nano-ferrites. NiFe_2_O_4_, ZnFe_2_O_4_, and CuFe_2_O_4_ nano-ferrites also demonstrated antibacterial activity, consistent with previous studies^23,29,30,34,46,80,115^.

Our findings suggest that combining Zn, Cu, and Ni into one structure enhance the synergetic effects. The small size in nanoparticles facilitates their penetration into bacterial cell membranes, leading to bacterial cell death. Additionally, the remarkable softness in the magnetization of Cu_0.15_Zn_0.2_Ni_0.65_Fe_2_O_4_ further increases their ability to penetrate bacterial cells. These properties highlight the potential for using Cu_0.15_Zn_0.2_Ni_0.65_Fe_2_O_4_ nanoparticles in biomedical applications.

## Conclusion

In this study, Cu_0.15_Zn_0.2_Ni_0.65_Fe_2_O_4_ nanoparticle was prepared through the co-precipitation technique. For comparison the antibacterial behavior, single-cation ferrites NiFe_2_O_4_, ZnFe_2_O_4_, and CuFe_2_O_4_ were also synthesized using the same procedure to enable a consistent evaluation of antibacterial activity. All ferrites were characterized using XRD and FTIR. Additional analyses including UV–Vis, SEM, EDX, XPS, AAS, TEM, and VSM were performed for the multicomponent Cu_0.15_Zn_0.2_Ni_0.65_Fe_2_O_4_ sample. XRD confirmed the formation of a cubic spinel phase for all ferrites. FTIR spectra revealed metal–oxygen vibrations associated with cation redistribution between tetrahedral and octahedral sites. SEM analysis showed aggregated nanoparticles with an average size of ~ 608 nm, while EDX confirmed elemental presence. AAS verified the availability of Cu^2+^, Zn^2+^, and Ni^2+^ ions, supporting their role in antibacterial activity. TEM images demonstrated spherical. VSM results indicated soft magnetic behavior with a saturation magnetization of ~ 54.3 emu/g. Antibacterial tests demonstrated that Cu_0.15_Zn_0.2_Ni_0.65_Fe_2_O_4_ exhibits stronger inhibitory activity against *S. aureus* and *E. coli*, at the concentration of 500 μg/ml obtained an inhibition zone ~ 20 mm for *S. aureus* with MIC equal 40 μg/ml, for *E. coli* ~ 17 mm with MIC equal 80 μg/ml and showing clear superiority over single-cation ferrites, enhanced performance arises from the synergistic effect of combining Cu, Zn, and Ni within a single spinel lattice. These results highlight the novelty of the Cu_0.15_Zn_0.2_Ni_0.65_Fe_2_O_4_ nanoparticles and its potential as an improved antibacterial nanomaterial for biomedical applications.

## Supplementary Information

Below is the link to the electronic supplementary material.

Supplementary Material 1

## Data Availability

The datasets generated during and/or analyzed during the current study are available from the corresponding author on reasonable request.
