# Supplementary material for: Structural, optical, and magnetic characterization of Cu–Zn–Ni spinel ferrite nanoparticles with antibacterial potential
Source: Sci Rep. 2026 Jan 22;16:3053. doi: 10.1038/s41598-025-34792-9 (PMC12830723; doi:10.1038/s41598-025-34792-9)
Supplement: Supplementary file 1 — Supplementary Material 1 [file 41598_2025_34792_MOESM1_ESM.pdf]

Ferrite-Evaluation report (Ferrite)

General information

Analysis date	2025-05-22 05:19:56	Measurement start time	2025-05-16 00:29:12
Analyst	Administrator	Operator	Administrator
Sample name	Ferrite	Comment	
Measured data name	C:\Users\Neoscope\Desktop\XRD DATA\16-5-2025\Dr.Samaa...	Memo	Ferrite

Measurement Conditions

X-Ray generator	40 kV, 15 mA	Scan mode	1D(scan)
Incident primary	No unit	Scan speed/Duration time	10.00 °/min
Goniometer	MiniFlex 300/600	Step width	0.015 °
Attachment	Standard sample stage	Scan axis	θ/2θ
Filter	None	Scan range	10 ~ 80 °
Selection slit	None	DS	1.25deg
Diffracted beam mono	None	IHS	10 mm
Detector	D/teX Ultra2	SS	Open
Optics attribute	None	RS	Open

Measured profile view

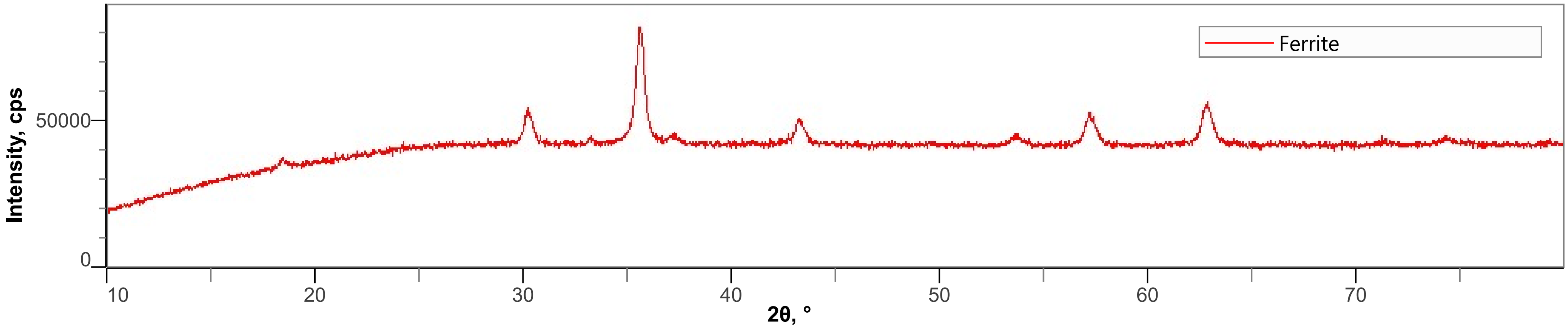

Peak profiling conditions

Peak search method	Second derivative method	σ cut	3.00		
Profile fitting	Run completed	Peak shape	Split pseudo-Voigt	Fitting condition	Auto(Refine background)

Peak Profile View

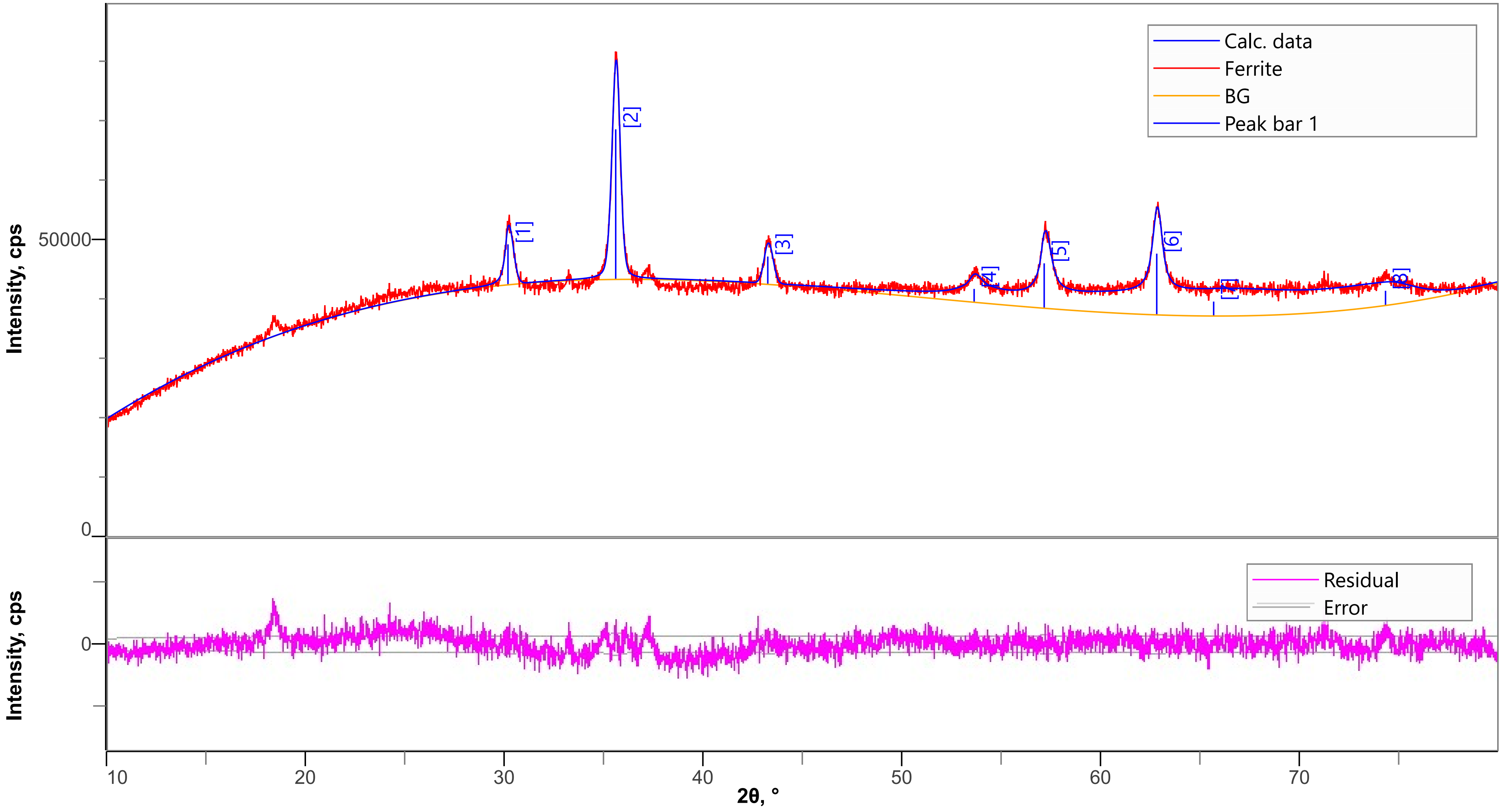

Qualitative Analysis Results

Phase name	Chemical formula	FOM	Phase reg. detail	Space Group	DB Card Number
Fe O (O H)	Fe H O2	0.435	S/M:COD	227 : Fd-3m:1	4344128
Franklinite	Cu0.5 Fe2 O4 Zn0.5	1.115	Import:COD	227 : Fd-3m:1	9012442
Fe2 Ni0.5 O4 Zn0.5	Zn.5Ni.5Fe2O4	1.203	Import:COD	227 : Fd-3m:1	9009920

Phase Data View

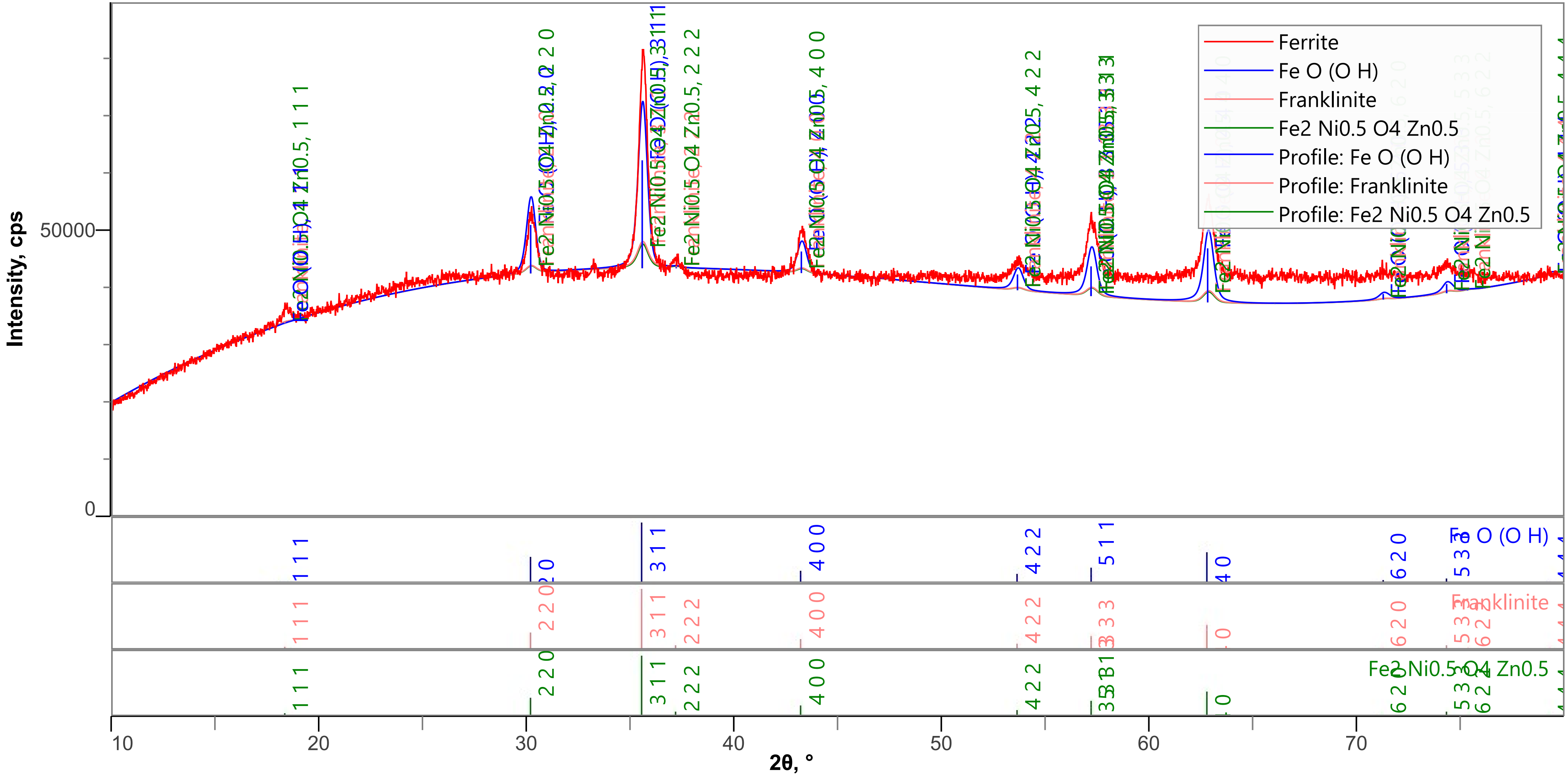

Peak list

No.	2θ, °	d, Å	Height, cps	FWHM, °	Int. I., cps°	Int. W., °	Asymmetry	Decay(ηL/mL)	Decay(ηH/mH)	Size, Å
1	30.185(6)	2.9584(5)	6802(99)	0.467(17)	3927(151)	0.58(3)	0.65(13)	0.90(11)	0.00(17)	184(7)
2	35.601(6)	2.5197(4)	25219(294)	0.477(5)	14416(168)	0.572(13)	1.08(6)	0.35(5)	0.34(5)	182.5(19)
3	43.25(3)	2.0903(12)	4680(70)	0.51(2)	2548(176)	0.54(5)	0.9(2)	0.0(3)	0.0(3)	175(8)
4	53.62(9)	1.708(3)	2243(36)	0.97(14)	4023(390)	1.8(2)	0.8(4)	1.3(3)	1.3(3)	96(14)
5	57.14(3)	1.6107(8)	7545(111)	0.57(3)	6813(247)	0.90(5)	0.7(2)	1.2(2)	0.89(15)	165(9)
6	62.80(3)	1.4784(6)	10289(145)	0.52(2)	8209(225)	0.80(3)	1.2(3)	0.93(14)	0.99(17)	188(8)
7	65.7(3)	1.421(5)	2423(39)	14.1(5)	36389(1612)	15.0(9)	2.2(3)	0.00(17)	0.0(10)	7.0(2)
8	74.30(12)	1.2755(17)	2467(39)	6.8(4)	25090(2433)	10.2(11)	3(2)	0.9(13)	0.79(16)	15.2(10)

No.	2θ, °	Phase Name	Chemical Formula	Card No	Norm. I.	Profile Type	Distributi...	Degree of Orientation
1	30.185(6)	Fe O (O H): 2 2 0,Franklinite: 2...	Fe H O2,Cu0.5 Fe2...	4344128,9012442,...	10.79	Split pseudo-Voigt	-	-
2	35.601(6)	Fe O (O H): 3 1 1,Franklinite: 3...	Fe H O2,Cu0.5 Fe2...	4344128,9012442,...	39.61	Split pseudo-Voigt	-	-
3	43.25(3)	Fe O (O H): 4 0 0,Franklinite: 4...	Fe H O2,Cu0.5 Fe2...	4344128,9012442,...	7.00	Split pseudo-Voigt	-	-
4	53.62(9)	Fe O (O H): 4 2 2,Franklinite: 4...	Fe H O2,Cu0.5 Fe2...	4344128,9012442,...	11.06	Split pseudo-Voigt	-	-
5	57.14(3)	Fe O (O H): 5 1 1,Franklinite: 5...	Fe H O2,Cu0.5 Fe2...	4344128,9012442,...	18.72	Split pseudo-Voigt	-	-
6	62.80(3)	Fe O (O H): 4 4 0,Franklinite: 4...	Fe H O2,Cu0.5 Fe2...	4344128,9012442,...	22.56	Split pseudo-Voigt	-	-
7	65.7(3)	Fe O (O H): 5 3 1,Franklinite: 5...	Fe H O2,Cu0.5 Fe2...	4344128,9012442,...	100.00	Split pseudo-Voigt	-	-
8	74.30(12)	Fe O (O H): 5 3 3,Franklinite: 5...	Fe H O2,Cu0.5 Fe2...	4344128,9012442,...	68.95	Split pseudo-Voigt	-	-

No.	2θ, °	Ring Factor	β Cluster
1	30.185(6)	-	-
2	35.601(6)	-	-
3	43.25(3)	-	-
4	53.62(9)	-	-
5	57.14(3)	-	-
6	62.80(3)	-	-
7	65.7(3)	-	-
8	74.30(12)	-	-

Lattice parameters

Phase name	a, Å	b, Å	c, Å	α, °	β, °	γ, °
Fe O (O H)	8.36290	8.36290	8.36290	90.000	90.000	90.000
Franklinite	8.36265	8.36265	8.36265	90.000	90.000	90.000
Fe2 Ni0.5 O4 Zn0.5	8.36273	8.36273	8.36273	90.000	90.000	90.000

d-I List

Fe O (O H)

No.	2θ, °	d, Å	h k l	Norm. I.
1	18.36006	4.82832	1 1 1	0.18
2	30.20220	2.95673	2 2 0	44.91
3	35.57534	2.52151	3 1 1	100.00
4	37.21390	2.41416	2 2 2	0.79
5	43.23830	2.09072	4 0 0	19.91
6	47.34310	1.91858	3 3 1	0.60
7	53.64644	1.70707	4 2 2	14.62
8	57.18951	1.60944	5 1 1	27.45
9	57.18951	1.60944	3 3 3	5.44
10	62.80490	1.47837	4 4 0	50.03
11	66.03882	1.41359	5 3 1	0.01
12	67.09841	1.39382	4 4 2	0.00
13	71.25981	1.32229	6 2 0	5.19
14	74.31353	1.27533	5 3 3	8.51
15	75.32091	1.26075	6 2 2	0.66
16	79.30781	1.20708	4 4 4	2.30

Franklinite

No.	2θ, °	d, Å	h k l	Norm. I.
1	18.36060	4.82818	1 1 1	6.34
2	30.20310	2.95664	2 2 0	30.01
3	35.57640	2.52144	3 1 1	100.00
4	37.21502	2.41409	2 2 2	7.64
5	43.23961	2.09066	4 0 0	18.71
6	47.34455	1.91852	3 3 1	0.40
7	53.64811	1.70702	4 2 2	10.09
8	57.19131	1.60939	5 1 1	24.73
9	57.19131	1.60939	3 3 3	6.20
10	62.80693	1.47832	4 4 0	40.98
11	66.04098	1.41355	5 3 1	0.70

Franklinite

No.	2θ, °	d, Å	h k l	Norm. I.
12	67.10061	1.39378	4 4 2	0.00
13	71.26219	1.32225	6 2 0	3.64
14	74.31605	1.27529	5 3 3	8.47
15	75.32347	1.26072	6 2 2	3.38
16	79.31056	1.20705	4 4 4	2.40

Fe2 Ni0.5 O4 Zn0.5

No.	2θ, °	d, Å	h k l	Norm. I.
1	18.36044	4.82822	1 1 1	6.82
2	30.20284	2.95667	2 2 0	31.78
3	35.57609	2.52146	3 1 1	100.00
4	37.21469	2.41411	2 2 2	7.10
5	43.23923	2.09068	4 0 0	18.18
6	47.34412	1.91854	3 3 1	0.16
7	53.64762	1.70703	4 2 2	10.48
8	57.19078	1.60941	5 1 1	26.44
9	57.19078	1.60941	3 3 3	5.71
10	62.80634	1.47833	4 4 0	41.07
11	66.04034	1.41356	5 3 1	0.67
12	67.09996	1.39379	4 4 2	0.00
13	71.26150	1.32226	6 2 0	3.72
14	74.31531	1.27530	5 3 3	8.47
15	75.32272	1.26073	6 2 2	3.25
16	79.30975	1.20706	4 4 4	2.24

Crystal Structure

Fe O (O H)

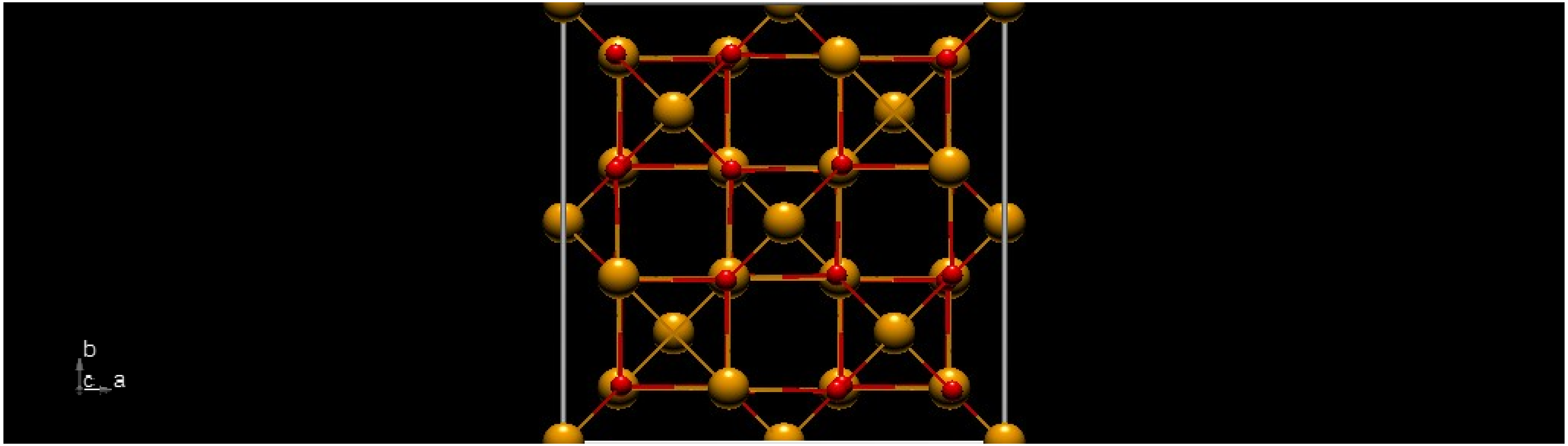

Structure parameters

Element	x	y	z	Occ.	B	Charge	Multiplicity	Valence	Bond Valence S...
Fe1(Fe)	0.000000	0.000000	0.000000	0.860	0.500	0	8	0.000	0.000
O1(O)	0.381000	0.381000	0.381000	1.000	0.500	0	32	0.000	0.000
Fe2(Fe)	0.625000	0.625000	0.625000	0.570	0.500	0	16	0.000	0.000

Distance

Bond Def.	Distance, Å
Fe2-Fe2(x,3/2-y,3/2-z)	2.95673
Fe2-Fe2(3/2-x,y,3/2-z)	2.95673
Fe2-Fe2(3/2-x,3/2-y,z)	2.95673
Fe2-Fe2(x,1-y,1-z)	2.95673
Fe2-Fe2(1-x,1-y,z)	2.95673

Distance

Bond Def.	Distance, Å
Fe2-Fe2(1-x,y,1-z)	2.95673
Fe1-O1(1/4-x,1/4-y,1/4-z)	1.89753
Fe1-O1(1/4-x,-1/4+y,-1/4+z)	1.89753
Fe1-O1(-1/4+x,1/4-y,-1/4+z)	1.89753
Fe1-O1(-1/4+x,-1/4+y,1/4-z)	1.89753
O1-Fe2(x,1-y,1-z)	2.04178
O1-Fe2(1-x,1-y,z)	2.04178
O1-Fe2(1-x,y,1-z)	2.04178

Franklinite

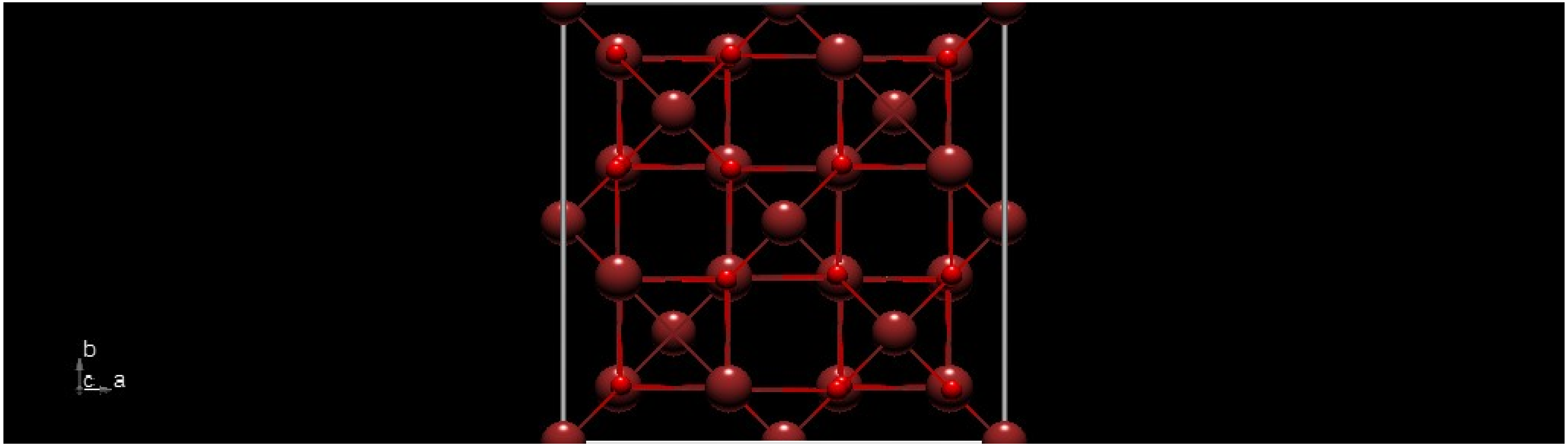

Structure parameters

Element	x	y	z	Occ.	B	Charge	Multiplicity	Valence	Bond Valence S...
Zn1(Zn)	0.000000	0.000000	0.000000	0.500	0.500	0	8	0.000	0.000
Fe1(Fe)	0.000000	0.000000	0.000000	0.500	0.500	0	8	0.000	0.000
Cu1(Cu)	0.625000	0.625000	0.625000	0.250	0.500	0	16	0.000	0.000

Structure parameters

Element	x	y	z	Occ.	B	Charge	Multiplicity	Valence	Bond Valence S...
Fe2(Fe)	0.625000	0.625000	0.625000	0.750	0.500	0	16	0.000	0.000
O1(O)	0.380000	0.380000	0.380000	1.000	0.500	0	32	0.000	0.000

Distance

Bond Def.	Distance, Å
Zn1-O1(1/4-x,1/4-y,1/4-z)	1.88299
Zn1-O1(1/4-x,-1/4+y,-1/4+z)	1.88299
Zn1-O1(-1/4+x,1/4-y,-1/4+z)	1.88299
Zn1-O1(-1/4+x,-1/4+y,1/4-z)	1.88299
Cu1-Cu1(3/2-x,3/2-y,z)	2.95664
Cu1-Cu1(x,1-y,1-z)	2.95664
Cu1-Cu1(1-x,1-y,z)	2.95664
Cu1-Cu1(1-x,y,1-z)	2.95664
Cu1-Cu1(x,3/2-y,3/2-z)	2.95664
Cu1-Cu1(3/2-x,y,3/2-z)	2.95664
Cu1-Fe2(3/2-x,3/2-y,z)	2.95664
Cu1-Fe2(x,1-y,1-z)	2.95664
Cu1-Fe2(1-x,1-y,z)	2.95664
Cu1-Fe2(1-x,y,1-z)	2.95664
Cu1-Fe2(x,3/2-y,3/2-z)	2.95664
Cu1-Fe2(3/2-x,y,3/2-z)	2.95664
Cu1-O1(x,1-y,1-z)	2.04970
Cu1-O1(1-x,1-y,z)	2.04970
Cu1-O1(1-x,y,1-z)	2.04970
Cu1-O1(5/4-x,1/4+y,1/4+z)	2.04970
Cu1-O1(1/4+x,1/4+y,5/4-z)	2.04970
Cu1-O1(1/4+x,5/4-y,1/4+z)	2.04970
Fe2-Fe2(x,3/2-y,3/2-z)	2.95664
Fe2-Fe2(3/2-x,y,3/2-z)	2.95664
Fe2-Fe2(3/2-x,3/2-y,z)	2.95664

Distance

Bond Def.	Distance, Å
Fe2-Fe2(x,1-y,1-z)	2.95664
Fe2-Fe2(1-x,1-y,z)	2.95664
Fe2-Fe2(1-x,y,1-z)	2.95664
Fe1-O1(1/4-x,1/4-y,1/4-z)	1.88299
Fe1-O1(1/4-x,-1/4+y,-1/4+z)	1.88299
Fe1-O1(-1/4+x,1/4-y,-1/4+z)	1.88299
Fe1-O1(-1/4+x,-1/4+y,1/4-z)	1.88299
Fe2-O1(x,1-y,1-z)	2.04970
Fe2-O1(1-x,1-y,z)	2.04970
Fe2-O1(1-x,y,1-z)	2.04970
Fe2-O1(5/4-x,1/4+y,1/4+z)	2.04970
Fe2-O1(1/4+x,1/4+y,5/4-z)	2.04970
Fe2-O1(1/4+x,5/4-y,1/4+z)	2.04970

Fe2 Ni0.5 O4 Zn0.5

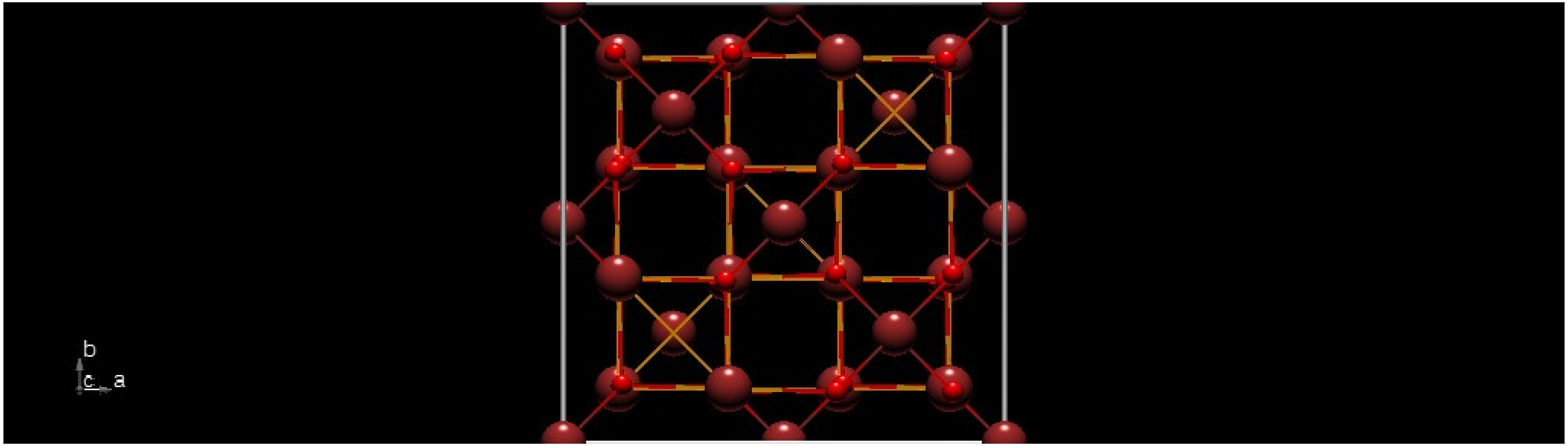

Structure parameters

Element	x	y	z	Occ.	B	Charge	Multiplicity	Valence	Bond Valence S...
Zn1(Zn)	0.000000	0.000000	0.000000	0.500	0.500	0	8	0.000	0.000
Fe1(Fe)	0.000000	0.000000	0.000000	0.500	0.500	0	8	0.000	0.000
Fe2(Fe)	0.625000	0.625000	0.625000	0.750	0.500	0	16	0.000	0.000
Ni1(Ni)	0.625000	0.625000	0.625000	0.250	0.500	0	16	0.000	0.000
O1(O)	0.382500	0.382500	0.382500	1.000	0.500	0	32	0.000	0.000

Distance

Bond Def.	Distance, Å
Zn1-O1(1/4-x,1/4-y,1/4-z)	1.91922
Zn1-O1(1/4-x,-1/4+y,-1/4+z)	1.91922
Zn1-O1(-1/4+x,1/4-y,-1/4+z)	1.91922
Zn1-O1(-1/4+x,-1/4+y,1/4-z)	1.91922
Ni1-Ni1(3/2-x,3/2-y,z)	2.95667
Ni1-Ni1(3/2-x,y,3/2-z)	2.95667
Ni1-Ni1(x,3/2-y,3/2-z)	2.95667
Ni1-Ni1(1-x,y,1-z)	2.95667
Ni1-Ni1(1-x,1-y,z)	2.95667
Ni1-Ni1(x,1-y,1-z)	2.95667
Ni1-O1(1-x,y,1-z)	2.02990
Ni1-O1(5/4-x,1/4+y,1/4+z)	2.02990
Ni1-O1(1/4+x,1/4+y,5/4-z)	2.02990
Ni1-O1(1/4+x,5/4-y,1/4+z)	2.02990
Ni1-O1(x,1-y,1-z)	2.02990
Ni1-O1(1-x,1-y,z)	2.02990
Fe2-Ni1(1-x,1-y,z)	2.95667
Fe2-Ni1(x,1-y,1-z)	2.95667
Fe2-Ni1(3/2-x,3/2-y,z)	2.95667
Fe2-Ni1(3/2-x,y,3/2-z)	2.95667
Fe2-Ni1(x,3/2-y,3/2-z)	2.95667
Fe2-Ni1(1-x,y,1-z)	2.95667

Distance

Bond Def.	Distance, Å
Fe2-Fe2(x,3/2-y,3/2-z)	2.95667
Fe2-Fe2(3/2-x,y,3/2-z)	2.95667
Fe2-Fe2(3/2-x,3/2-y,z)	2.95667
Fe2-Fe2(x,1-y,1-z)	2.95667
Fe2-Fe2(1-x,1-y,z)	2.95667
Fe2-Fe2(1-x,y,1-z)	2.95667
Fe1-O1(1/4-x,1/4-y,1/4-z)	1.91922
Fe1-O1(1/4-x,-1/4+y,-1/4+z)	1.91922
Fe1-O1(-1/4+x,1/4-y,-1/4+z)	1.91922
Fe1-O1(-1/4+x,-1/4+y,1/4-z)	1.91922
Fe2-O1(1/4+x,5/4-y,1/4+z)	2.02990
Fe2-O1(x,1-y,1-z)	2.02990
Fe2-O1(1-x,1-y,z)	2.02990
Fe2-O1(1-x,y,1-z)	2.02990
Fe2-O1(5/4-x,1/4+y,1/4+z)	2.02990
Fe2-O1(1/4+x,1/4+y,5/4-z)	2.02990

Commander Sample ID (Coupled TwoTheta/Theta)

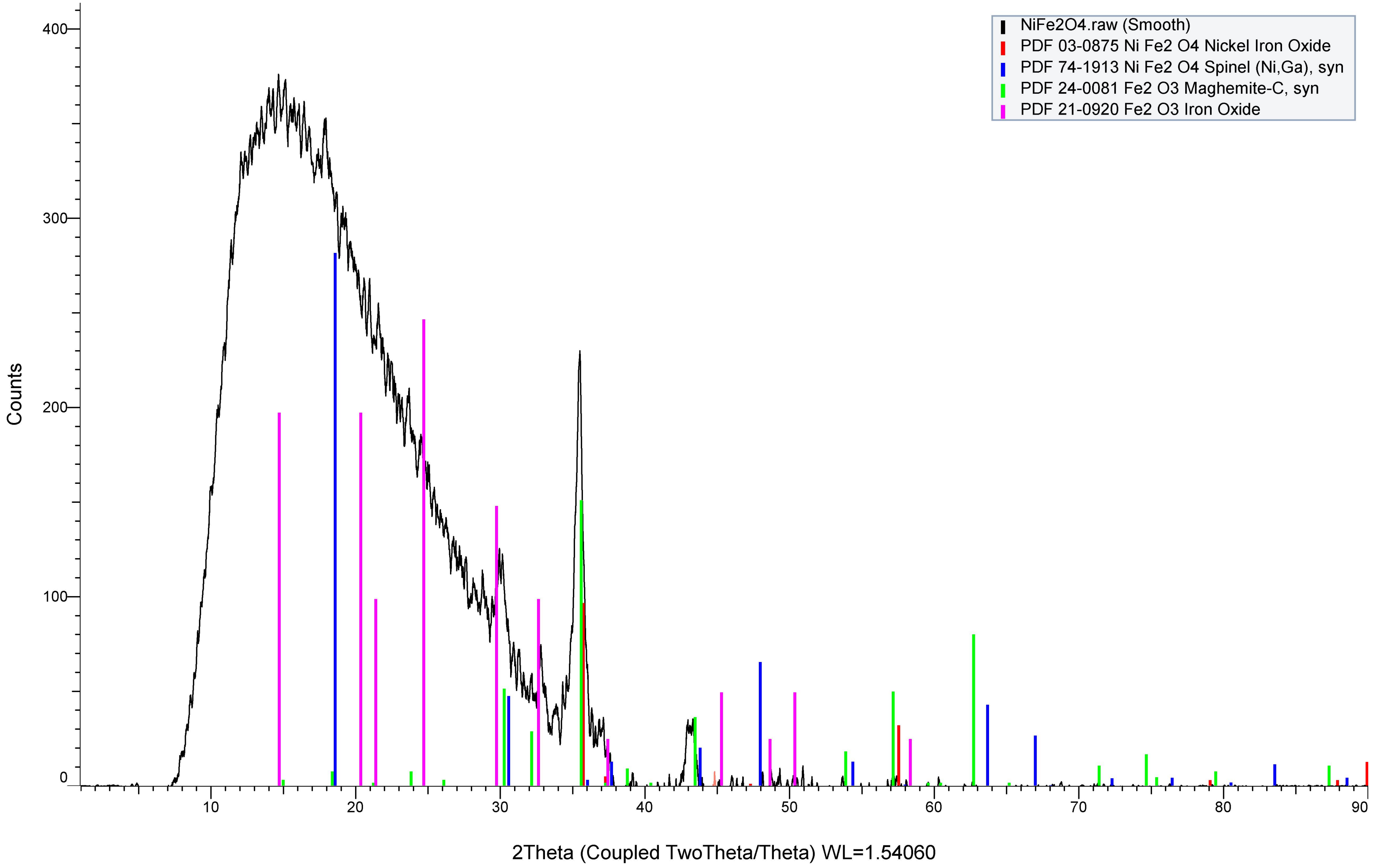

2Theta

Show	Icon	Color	Index	Name	Parent	Scan	Pattern #
Yes			0	PDF 03-0875	Pattern List #3	NiFe2O4.raw (Smooth) #1	PDF 03-0875
Yes			1	PDF 74-1913	Pattern List #3	NiFe2O4.raw (Smooth) #1	PDF 74-1913
Yes			2	PDF 24-0081	Pattern List #3	NiFe2O4.raw (Smooth) #1	PDF 24-0081
Yes			3	PDF 21-0920	Pattern List #3	NiFe2O4.raw (Smooth) #1	PDF 21-0920

Compound Name	Formula	Quality	Y-Scale	I/Ic DB	I/Ic User	S-Q	Added Reference	d x by
Nickel Iron Oxide	Ni Fe2 O4	Blank	25.6825 %					1.0000
Spinel (Ni,Ga), syn	Ni Fe2 O4	Calculated	74.8735 %	9.920				1.0000
Maghemite-C, syn	Fe2 O3	Blank	40.1168 %					1.0000
Iron Oxide	Fe2 O3	Blank	65.5362 %					1.0000

Scan WL	Wavelength	System	Space Group	a	b	c	alpha	beta	gamma	Z	Volume
Yes	1.54060	Cubic		8.34000							580.09
Yes	1.54060	Cubic	Fd-3m (227)	8.25800						8	563.15
Yes	1.54060	Cubic	P4232 (208)	8.35000						11	582.18
Yes	1.54060										0.00

Density	Cell Tuned	F (N)
	No	F17= 3.9(0.0690, 64)
6.183	No	F17= 999.9(0.0000, 17)
4.899	No	F28= 5.5(0.0920, 55)
	No	

Pattern: PDF 03-0875 Radiation: 1.54060 Quality: Blank

**Formula**	Ni Fe2 O4
**Name**	Nickel Iron Oxide
**Name (mineral)**	
**Name (common)**	

**Lattice:**	Cubic
**S.G.:**	
**Mol. weight =**	234.39
**Volume [CD] =**	580.09
**Dx =**	
**Dm =**	
**I/Icor =**	

a =	8.34000	alpha =	
b =		beta =	
c =		gamma =	
a/b =	1.00000	Z =	
c/b =	1.00000		

d	2θ	I	h	k	l
4.82000	18.392	7	1	1	1
2.95000	30.273	20	2	2	0
2.51000	35.744	100	3	1	1
2.41000	37.281	5	2	2	2
2.08000	43.473	23	4	0	0
1.92000	47.306	1	3	3	1
1.70000	53.888	13	4	2	2
1.60000	57.559	33	5	1	1
1.48000	62.728	53	4	4	0
1.32000	71.403	4	6	2	0
1.27000	74.679	9	5	3	3
1.21000	79.079	3	4	4	4
1.11000	87.889	3	6	4	2
1.09000	89.934	13	7	3	1
1.04000	95.578	4	8	0	0
0.98200	103.334	1	6	6	0
0.96200	106.399	5	7	5	1

Deleted By or Rejected By: Deleted by NBS card Set 10
Sample Preparation: Prepared by fusion at precipitated hydroxides

Primary Reference
Publication: Private Communication
Authors: Dow Chemical Company, Midland, MI, USA.

**Radiation:**	MoKa1
**Wavelength:**	1.54060
**SS/FOM:**	3.9 (0.069,64)
**Filter:**	F
**d-spacing:**	

Pattern: PDF 74-1913   Radiation: 1.54060   Quality: Calculated

**Formula**	Ni Fe2 O4
**Name**	Nickel Iron Oxide
**Name (mineral)**	Spinel (Ni,Ga), syn
**Name (common)**	

**Lattice:**	Cubic	**Mol. weight =**	234.39
**S.G.:**	Fd-3m (227)	**Volume [CD] =**	563.15
		**Dx =**	
		**Dm =**	
		**I/Icor =**	9.920

**a =**	8.25800	**alpha =**	
**b =**		**beta =**	
**c =**		**gamma =**	
**a/b =**	1.00000	**Z =**	8
**c/b =**	1.00000		

ICSD Collection Code: 027903
Remark From ICSD/CSD: REM MAG
Test From ICSD: No R value given
Test From ICSD: At least one TF missing
Sample Preparation: Prepared from oxides at 1673 K for 1-2 hours
Additional Pattern: See PDF 10-114
Test From ICSD: Calc. density unusual but tolerable
Article Title: Cation Distribution and g-Factors of Certain Spinels Containing Ni(II), Mn(II), Co(II), Al(III), Ga(III), and Fe(III)

Structure
Publication: J. Chem. Phys.
Detail: volume 22, page 1597 (1954)
Authors: Greenwald, S., Pickart, S.J., Grannis, F.H.
Primary Reference
Publication: Calculated from ICSD using POWD-12++

**Radiation:**	CuKa1	**Filter:**	Not specified
**Wavelength:**	1.54060	**d-spacing:**	
**SS/FOM:**	999.9 (0,17)		

d	2θ	I	h	k	l
4.76776	18.595	999	1	1	1
2.91964	30.595	168	2	2	0
2.48988	36.043	11	3	1	1
2.38388	37.704	45	2	2	2
2.06450	43.816	71	4	0	0
1.89452	47.982	232	3	3	1
1.68566	54.384	45	4	2	2
1.58925	57.985	2	5	1	1
1.45982	63.696	152	4	4	0
1.39586	66.988	94	5	3	1
1.37633	68.067	1	4	4	2
1.30570	72.307	14	6	2	0
1.25933	75.421	1	5	3	3
1.24494	76.449	15	6	2	2
1.19194	80.520	6	4	4	4
1.15635	83.541	40	7	1	1
1.10352	88.540	15	6	4	2

Pattern: PDF 24-0081 Radiation: 1.54060 Quality: Blank

**Formula**	Fe2 O3
**Name**	Iron Oxide
**Name (mineral)**	Maghemite-C, syn
**Name (common)**	

**Lattice:**	Cubic
**S.G.:**	P4232 (208)
**Mol. weight =**	159.69
**Volume [CD] =**	582.18
**Dx =**	
**Dm =**	4.899
**I/Icor =**	

**a =**	8.35000	**alpha =**	
**b =**		**beta =**	
**c =**		**gamma =**	
**a/b =**	1.00000	**Z =**	11
**c/b =**	1.00000		

Color: Brown
General Comments: Low angle lines calculated from cell dimensions
Additional Pattern: To replace 4-755
General Comments: Optical data on specimen from Iron Mountain, Shasta County, California, USA
General Comments: Measured density from Kumano mine, Yamaguchi Prefecture, Japan
General Comments: Opaque mineral optical data on specimen from Gara Djebilet, Algeria: RR#2R#e=24.6, Disp.=16, VHN#5#0=412, Color values=.293, .304, 24.1, Ref
: IMA Commission on Ore Microscopy QDF
Optical Data: B=2.74
Deleted By or Rejected By: Deleted by 39-1346

Primary Reference
Publication: Z. Phys. Chem.
Detail: volume 44, page 216 (1939)
Authors: Haul, Shoon.

**Radiation:**	FeKa	**Filter:**	Not specified
**Wavelength:**	1.54060	**d-spacing:**	
**SS/FOM:**	5.5 (0.092,55)		

d	2θ	I	h	k	l
5.90000	15.004	2	1	1	0
4.82000	18.392	5	1	1	1
4.18000	21.239	1	2	0	0
3.73000	23.836	5	2	1	0
3.41000	26.111	2	2	1	1
2.95000	30.273	34	2	2	0
2.78000	32.173	19	2	2	1
2.52000	35.598	100	3	1	1
2.41000	37.281	1	2	2	2
2.32000	38.784	6	3	2	0
2.23000	40.416	1	3	2	1
2.08000	43.473	24	4	0	0
1.87000	48.652	1	4	2	0
1.70000	53.888	12	4	2	2
1.61000	57.168	33	5	1	1
1.55000	59.599	1	5	2	0
1.53000	60.459	1	5	2	1
1.48000	62.728	53	4	4	0
1.43000	65.186	1	5	3	0
1.32000	71.403	7	6	2	0
1.27000	74.679	11	5	3	3
1.26000	75.374	3	6	2	2
1.20500	79.472	5	4	4	4
1.11600	87.297	7	6	4	2
1.08700	90.250	19	7	3	1
1.06900	92.205	1	6	5	0
1.04400	95.095	9	8	0	0
1.02800	97.063	1	7	4	1

Pattern: PDF 21-0920 Radiation: 1.54060 Quality: Blank

**Formula**	Fe_2O_3
**Name**	Iron Oxide
**Name (mineral)**	
**Name (common)**	

d	2θ	I	h	k	l
6.01000	14.728	80			
4.36000	20.352	80			
4.15000	21.394	40			
3.60000	24.710	100			
3.00000	29.757	60			
2.74000	32.655	40			
2.40000	37.442	10			
2.00000	45.306	20			
1.87000	48.652	10			
1.81000	50.375	20			
1.58000	58.357	10			

Lattice:
S.G.:

Mol. weight = 159.69
Volume [CD] = 0
Dx =
Dm =
I/Icor =

a =
b =
c =
a/b =
c/b =

alpha =
beta =
gamma =
Z =

Primary Reference
Publication: Bull. Soc. Chim. Fr.
Authors: Walter-Levy, Quemeneur.

Radiation: CuKa1
Wavelength: 1.54060
SS/FOM:

Filter: Not specified
d-spacing:

Commander Sample ID (Coupled TwoTheta/Theta)

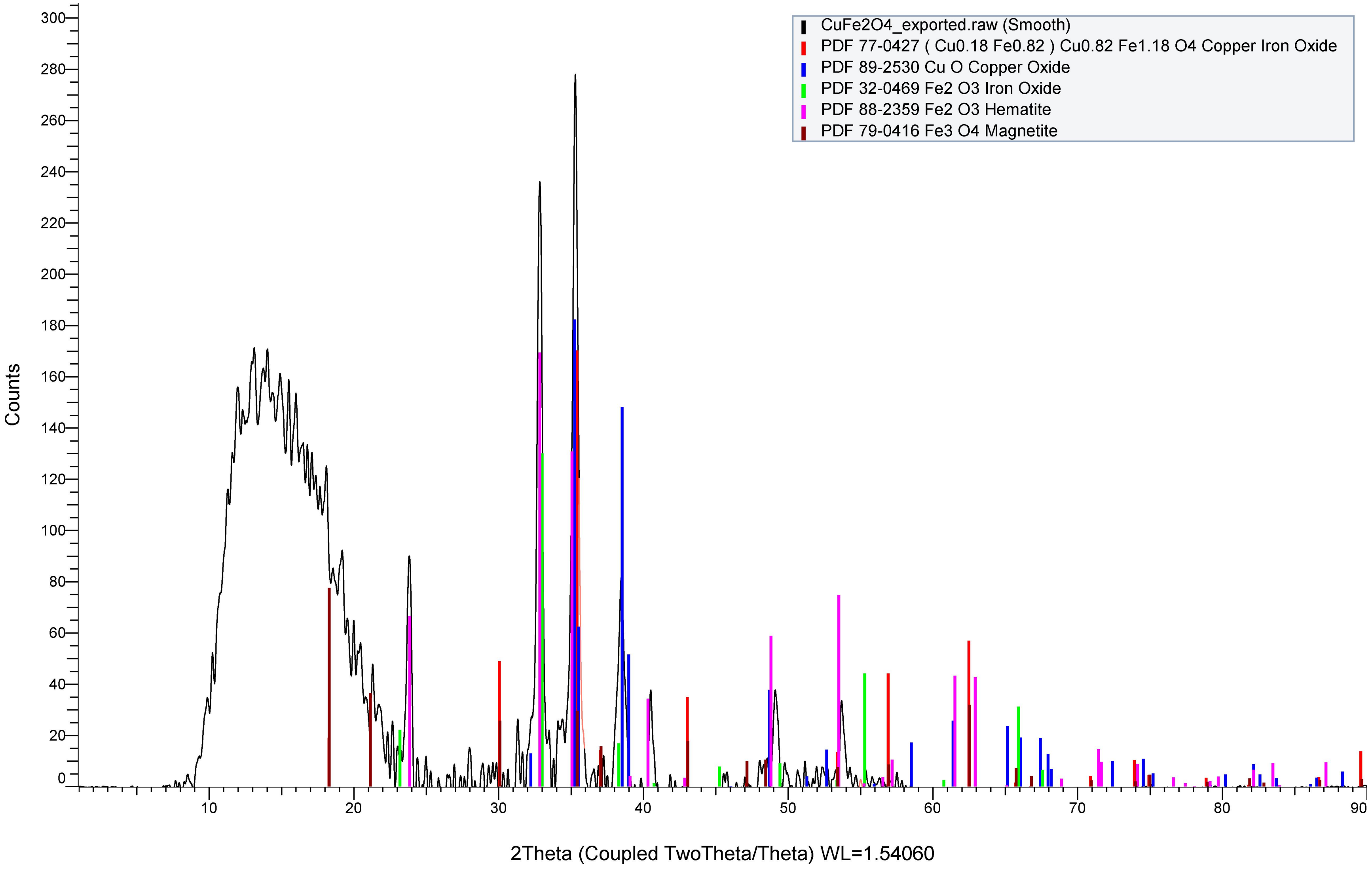

2Theta

Show	Icon	Color	Index	Name	Parent	Scan	Pattern #
Yes			0	PDF 77-0427	Pattern List #3	CuFe2O4_exported.raw (Smooth) #1	PDF 77-0427
Yes			1	PDF 89-2530	Pattern List #3	CuFe2O4_exported.raw (Smooth) #1	PDF 89-2530
Yes			2	PDF 32-0469	Pattern List #3	CuFe2O4_exported.raw (Smooth) #1	PDF 32-0469
Yes			3	PDF 88-2359	Pattern List #3	CuFe2O4_exported.raw (Smooth) #1	PDF 88-2359
Yes			4	PDF 79-0416	Pattern List #3	CuFe2O4_exported.raw (Smooth) #1	PDF 79-0416

Compound Name	Formula	Quality	Y-Scale	I/Ic DB	I/Ic User	S-Q
Copper Iron Oxide	(Cu0.18 Fe0.82) Cu0.82 Fe1.18 O4	Calculated	61.2354 %	5.140		
Copper Oxide	Cu O	Calculated	65.5844 %	3.690		
Iron Oxide	Fe2 O3	Calculated	46.7855 %			
Hematite	Fe2 O3	Hypothetical	60.9565 %	2.930		
Magnetite	Fe3 O4	Calculated	27.8879 %	3.840		

Added Reference	d x by	Scan WL	Wavelength	System	Space Group	a	b
	1.0000	Yes	1.54060	Cubic	Fd-3m (227)	8.40000	
	1.0000	Yes	1.54060	Monoclinic	C2/c (15)	4.68390	3.47340
	1.0000	Yes	1.54060	Cubic	Ia-3 (206)	9.39300	
	1.0000	Yes	1.54060	Rhombo.H.axes	R-3c (167)	5.11200	
	1.0000	Yes	1.54060	Cubic	F-43m (216)	8.39410	

c	alpha	beta	gamma	Z	Volume	Density	Cell Tuned	F (N)
				8	592.70	5.361	No	F18= 999.9(0.0001, 18)
5.12260		99.730		4	82.14	6.431	No	F29= 205.5(0.0040, 35)
				16	828.73	5.119	No	F12= 47.9(0.0104, 24)
13.82000				6	312.77	5.086	No	F29= 999.9(0.0002, 30)
				8	591.46	5.199	No	F21= 999.9(0.0001, 21)

Pattern: PDF 77-0427 Radiation: 1.54060 Quality: Calculated

**Formula**	(Cu0.18 Fe0.82) Cu0.82 Fe1.18 O4
**Name**	Copper Iron Oxide
**Name (mineral)**	
**Name (common)**	

**Lattice:**	Cubic	**Mol. weight =**	239.24
**S.G.:**	Fd-3m (227)	**Volume [CD] =**	592.7
		**Dx =**	
		**Dm =**	
		**I/Icor =**	5.140

**a =**	8.40000	**alpha =**	
**b =**		**beta =**	
**c =**		**gamma =**	
**a/b =**	1.00000	**Z =**	8
**c/b =**	1.00000		

ICSD Collection Code: 039132
Remark From ICSD/CSD: REM J OXYGEN ATOM COORDINATES WERE READ FROM THE DIAGRAM
Test From ICSD: At least one TF missing
Test From ICSD: No R value given
Additional Pattern: See PDF 77-10
Article Title: On crystalline structure of cupric ferrite

Structure
Publication: Denki Kagaku (Electrochemistry)
Detail: volume 23, page 81 (1955)
Authors: Inoue, T.
Primary Reference
Publication: Calculated from ICSD using POWD-12++

**Radiation:**	CuKa1	**Filter:**	Not specified
**Wavelength:**	1.54060	**d-spacing:**	
**SS/FOM:**	999.9 (0.0001,18)		

d	2θ	I	h	k	l
4.84974	18.278	112	1	1	1
2.96985	30.066	287	2	2	0
2.53270	35.413	999	3	1	1
2.42487	37.044	84	2	2	2
2.10000	43.038	205	4	0	0
1.92709	47.122	7	3	3	1
1.71464	53.391	79	4	2	2
1.61658	56.914	259	5	1	1
1.48492	62.497	334	4	4	0
1.41986	65.710	9	5	3	1
1.40000	66.763	1	4	4	2
1.32816	70.898	24	6	2	0
1.28099	73.931	61	5	3	3
1.26635	74.931	26	6	2	2
1.21244	78.889	20	4	4	4
1.17624	81.822	4	5	5	1
1.12250	86.666	22	6	4	2
1.09359	89.559	81	7	3	1

Pattern: PDF 89-2530 Radiation: 1.54060 Quality: Calculated

**Formula**	Cu O
**Name**	Copper Oxide
**Name (mineral)**	
**Name (common)**	

**Lattice:**	Monoclinic	**Mol. weight =**	79.55
**S.G.:**	C2/c (15)	**Volume [CD] =**	82.14
		**Dx =**	
		**Dm =**	
		**I/Icor =**	3.690

**a =**	4.68390	**alpha =**	
**b =**	3.47340	**beta =**	99.730
**c =**	5.12260	**gamma =**	
**a/b =**	1.34851	**Z =**	4
**c/b =**	1.47481		

ICSD Collection Code: 043180
Remark From ICSD/CSD: REM M Mechanically induced reduction of CuO by Fe, measureme
Remark From ICSD/CSD: REM milling CuO with Fe for 25 min, R(Bragg)=.0038
Article Title: A crystallographic contribution to the mechanism of a mechanically induced solid state reaction

Structure
Publication: J. Solid State Chem.
Detail: volume 122, page 273 (1996)
Authors: Calos, N.J., Forrester, J.S., Schaffer, G.B.
Primary Reference
Publication: Calculated from ICSD using POWD-12++

**Radiation:**	CuKa1	**Filter:**	Not specified
**Wavelength:**	1.54060	**d-spacing:**	
**SS/FOM:**	205.5 (0.004,35)		

d	2θ	I	h	k	l
2.77554	32.226	71	1	1	0
2.54381	35.253	999	-1	1	1
2.52446	35.533	342	0	0	2
2.33419	38.538	812	1	1	1
2.30826	38.989	283	2	0	0
1.96981	46.040	1	-1	1	2
1.86796	48.708	207	-2	0	2
1.77969	51.294	22	1	1	2
1.73670	52.660	79	0	2	0
1.64226	55.945	6	0	2	1
1.57601	58.519	94	2	0	2
1.50865	61.406	141	-1	1	3
1.43081	65.145	130	0	2	2
1.41294	66.073	105	-3	1	1
1.38777	67.430	104	2	2	0
1.37833	67.955	70	1	1	3
1.37430	68.181	38	-2	2	1
1.31866	71.486	2	-3	1	2
1.30420	72.403	55	3	1	1
1.30420	72.403	55	2	2	1
1.27191	74.548	59	-2	2	2
1.26223	75.218	28	0	0	4
1.20859	79.190	4	0	2	3
1.19580	80.207	26	-1	1	4
1.19580	80.207	26	-2	0	4
1.17224	82.161	48	-3	1	3
1.16709	82.602	26	2	2	2
1.15413	83.738	18	4	0	0
1.12846	86.096	5	-2	2	3
1.12392	86.529	19	1	3	0
1.12392	86.529	19	-4	0	2
1.10591	88.299	32	-1	3	1

Pattern: PDF 32-0469 Radiation: 1.54060 Quality: Calculated

**Formula**	Fe2 O3
**Name**	Iron Oxide
**Name (mineral)**	
**Name (common)**	

**Lattice:**	Cubic	**Mol. weight =**	159.69
**S.G.:**	Ia-3 (206)	**Volume [CD] =**	828.73
		**Dx =**	
		**Dm =**	
		**I/Icor =**	

**a =**	9.39300	**alpha =**	
**b =**		**beta =**	
**c =**		**gamma =**	
**a/b =**	1.00000	**Z =**	16
**c/b =**	1.00000		

General Comments: Diffraction samples-thin films prepared by vapor deposition on polycrystalline alumina substrated in the presence of dry and wet oxygen gas (evaporator 180 C, reactor 300 C)
Structure: Isostructural with α-2 O3bixbyite structure). Lattice parameter determined from Guinier pattern α-2 O3nternal standard
General Comments: Calculated pattern agrees substantially with reflections and intensities determined by Guinier and diffractometer methods by authors
Additional Pattern: To replace 19-615
Deleted By or Rejected By: Deleted by 39-238

Primary Reference
Publication: J. Electrochem. Soc.
Detail: volume 124, page 451 (1977)
Authors: Ben-Dor, L. et al.

**Radiation:**	CuKa1	**Filter:**	Not specified
**Wavelength:**	1.54060	**d-spacing:**	
**SS/FOM:**	47.9 (0.0104,24)		

d	2θ	I	h	k	l
3.83400	23.181	17	2	1	1
2.71100	33.015	100	2	2	2
2.34800	38.303	13	4	0	0
2.21300	40.740	1	4	1	1
2.00200	45.258	6	3	3	2
1.84200	49.440	7	4	3	1
1.71500	53.379	2	5	2	1
1.66000	55.296	34	4	4	0
1.52300	60.766	2	6	1	1
1.41600	65.912	24	6	2	2
1.38500	67.583	5	6	3	1
1.27800	74.133	4	7	2	1

Pattern: PDF 88-2359 Radiation: 1.54060 Quality: Hypothetical

**Formula**	Fe_2O_3
**Name**	Iron Oxide
**Name (mineral)**	Hematite
**Name (common)**	

**Lattice:**	Rhombo.H.axes	**Mol. weight =**	159.69
**S.G.:**	R-3c (167)	**Volume [CD] =**	312.77
		**Dx =**	
		**Dm =**	
		**I/Icor =**	2.930

**a =**	5.11200	**alpha =**	
**b =**		**beta =**	
**c =**	13.82000	**gamma =**	
**a/b =**	1.00000	**Z =**	6
**c/b =**	2.70344		

ICSD Collection Code: 041541
Hypothetical Structure: Structure calculated theoretically
Remark From ICSD/CSD: REM M Ab initio all-electron (AE) calculations
Remark From ICSD/CSD: REM DEN
Remark From ICSD/CSD: REM M PDF 33-664
Test From ICSD: No R value given
Test From ICSD: At least one TF missing
Article Title: Theoretical study of electronic, magnetic, and structural properties of alpha-Fe2 O3 (hematite)

Structure
Publication: Phys. Rev. B: Condens. Matter
Detail: volume 51, page 7441 (1995)
Authors: Catti, M., Valerio, G., Dovesi, R.
Primary Reference
Publication: Calculated from ICSD using POWD-12++

**Radiation:**	CuKa1	**Filter:**	Not specified
**Wavelength:**	1.54060	**d-spacing:**	
**SS/FOM:**	999.9 (0.0002,30)		

d	2θ	I	h	k	l
3.72768	23.851	392	0	1	2
2.72372	32.856	999	1	0	4
2.55600	35.080	771	1	1	0
2.30333	39.076	24	0	0	6
2.23502	40.321	202	1	1	3
2.10804	42.866	20	2	0	2
1.86384	48.823	347	0	2	4
1.71108	53.511	441	1	1	6
1.66116	55.254	7	2	1	1
1.62629	56.544	22	1	2	2
1.60932	57.194	62	0	1	8
1.50597	61.527	255	2	1	4
1.47571	62.931	252	3	0	0
1.43142	65.114	3	1	2	5
1.36186	68.891	18	2	0	8
1.31922	71.451	86	1	0	10
1.31628	71.636	57	1	1	9
1.27800	74.133	52	2	2	0
1.24256	76.622	21	0	3	6
1.23149	77.438	8	2	2	3
1.22304	78.074	1	1	3	1
1.20893	79.163	13	3	1	2
1.20190	79.718	23	1	2	8
1.17228	82.157	39	0	2	10
1.15697	83.486	54	1	3	4
1.15167	83.957	3	0	0	12
1.12212	86.702	1	3	1	5
1.11751	87.149	56	2	2	6
1.09285	89.636	6	0	4	2

Pattern: PDF 79-0416 Radiation: 1.54060 Quality: Calculated

**Formula**	Fe3 O4
**Name**	Iron Oxide
**Name (mineral)**	Magnetite
**Name (common)**	

**Lattice:**	Cubic	**Mol. weight =**	231.54
**S.G.:**	F-43m (216)	**Volume [CD] =**	591.46
		**Dx =**	
		**Dm =**	
		**I/Icor =**	3.840

**a =**	8.39410	**alpha =**	
**b =**		**beta =**	
**c =**		**gamma =**	
**a/b =**	1.00000	**Z =**	8
**c/b =**	1.00000		

ICSD Collection Code: 065338
Temperature Factor: ATF
Test From ICSD: At least one TF implausible
Sample Source Or Locality: Specimen from magnetite chlorite schist, Dana Collection 633
Additional Pattern: See PDF 19-629
Remark From ICSD/CSD: R(Fd3-m)=0.025, cp. 49549
Test From ICSD: Calc. density unusual but tolerable
Article Title: The structure of magnetite: Symmetry of cubic spinels

Structure
Publication: J. Solid State Chem.
Detail: volume 62, page 75 (1986)
Authors: Fleet, M.E.
Primary Reference
Publication: Calculated from ICSD using POWD-12++

**Radiation:**	CuKa1	**Filter:**	Not specified
**Wavelength:**	1.54060	**d-spacing:**	
**SS/FOM:**	999.9 (0.0001,21)		

d	2θ	I	h	k	l
4.84634	18.291	999	1	1	1
4.19705	21.151	470	2	0	0
2.96776	30.087	332	2	2	0
2.53092	35.439	381	3	1	1
2.42317	37.071	203	2	2	2
2.09853	43.070	230	4	0	0
1.92574	47.157	129	3	3	1
1.87698	48.459	135	4	2	0
1.71344	53.431	98	4	2	2
1.61545	56.958	112	3	3	3
1.48388	62.545	411	4	4	0
1.41886	65.763	93	5	3	1
1.39902	66.816	53	4	4	2
1.32722	70.955	32	6	2	0
1.28009	73.991	26	5	3	3
1.26546	74.993	60	6	2	2
1.21158	78.956	24	4	4	4
1.17541	81.892	41	5	5	1
1.16405	82.866	20	6	4	0
1.12171	86.742	33	6	4	2
1.09282	89.639	37	7	3	1

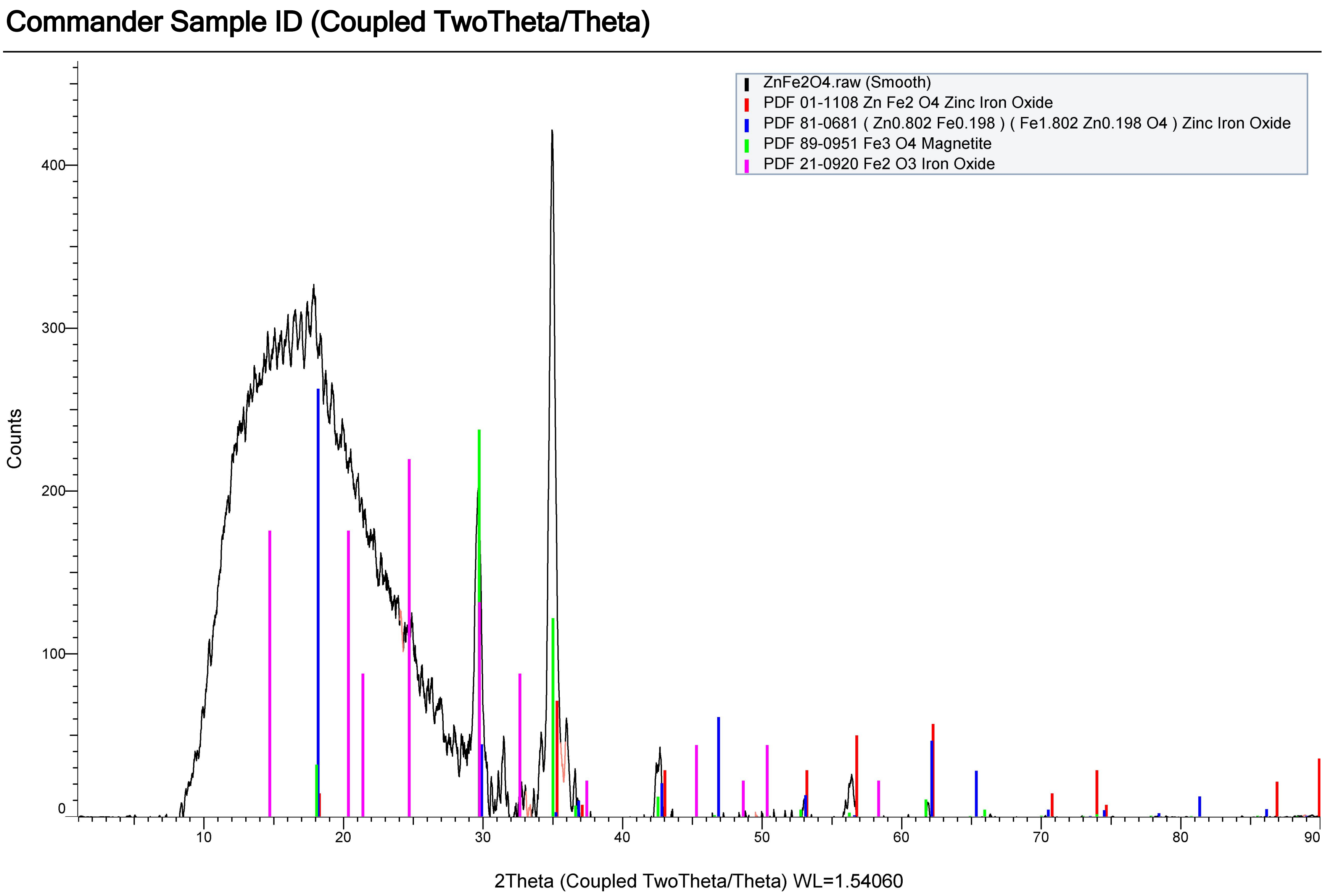

Commander Sample ID (Coupled TwoTheta/Theta)
ZnFe2O4.raw (Smooth)
PDF 01-1108 Zn Fe2 O4 Zinc Iron Oxide
PDF 81-0681 (Zn0.802 Fe0.198) (Fe1.802 Zn0.198 O4) Zinc Iron Oxide
PDF 89-0951 Fe3 O4 Magnetite
PDF 21-0920 Fe2 O3 Iron Oxide
Counts
400
300
200
100
0
10
20
30
40
50
60
70
80
90
2Theta (Coupled TwoTheta/Theta) WL=1.54060

2Theta

Show	Icon	Color	Index	Name	Parent	Scan	Pattern #
Yes			0	PDF 01-1108	Pattern List #3	ZnFe2O4.raw (Smooth) #1	PDF 01-1108
Yes			1	PDF 81-0681	Pattern List #3	ZnFe2O4.raw (Smooth) #1	PDF 81-0681
Yes			2	PDF 89-0951	Pattern List #3	ZnFe2O4.raw (Smooth) #1	PDF 89-0951
Yes			3	PDF 21-0920	Pattern List #3	ZnFe2O4.raw (Smooth) #1	PDF 21-0920

Compound Name	Formula	Quality	Y-Scale	I/Ic DB	I/Ic User	S-Q
Zinc Iron Oxide	Zn Fe2 O4	Blank	16.8698 %			
Zinc Iron Oxide	(Zn0.802 Fe0.198) (Fe1.802 Zn0.198 O4)	Calculated	62.3032 %	9.570		
Magnetite	Fe3 O4	Calculated	56.3575 %	1.180		
Iron Oxide	Fe2 O3	Blank	52.0614 %			

Added Reference	d x by	Scan WL	Wavelength	System	Space Group	a	b	c	alpha
	1.0000	Yes	1.54060	Cubic	Fd-3m (227)	8.43000			
	1.0000	Yes	1.54060	Cubic	Fd-3m (227)	8.43990			
	1.0000	Yes	1.54060	Cubic	Fd-3m (227)	8.49100			
	1.0000	Yes	1.54060						

beta	gamma	Z	Volume	Density	Cell Tuned	F (N)
		8	599.08	5.249	No	F19= 5.7(0.0970, 34)
		8	601.19	5.326	No	F17= 999.9(0.0002, 18)
		8	612.18	5.023	No	F18= 999.9(0.0001, 18)
			0.00		No	

Pattern: PDF 01-1108 Radiation: 1.54060 Quality: Blank

**Formula**	Zn Fe2 O4
**Name**	Zinc Iron Oxide
**Name (mineral)**	
**Name (common)**	

**Lattice:**	Cubic
**S.G.:**	Fd-3m (227)
**Mol. weight =**	241.07
**Volume [CD] =**	599.08
**Dx =**	
**Dm =**	5.249
**I/Icor =**	

a =	8.43000	alpha =	
b =		beta =	
c =		gamma =	
a/b =	1.00000	Z =	8
c/b =	1.00000		

d	2θ	I	h	k	l
4.85000	18.277	20	1	1	1
2.99000	29.858	50	2	2	0
2.54000	35.308	100	3	1	1
2.42000	37.121	10	2	2	2
2.10000	43.038	40	4	0	0
1.72000	53.211	40	4	2	2
1.62000	56.783	70	5	1	1
1.49000	62.260	80	4	4	0
1.33000	70.785	20	6	2	0
1.28000	73.997	40	5	3	3
1.27000	74.679	10	6	2	2
1.12000	86.907	30	6	4	2
1.09000	89.934	50	7	3	1
1.05000	94.381	30	8	0	0
0.97000	105.145	40	6	6	2
0.88000	122.171	20	9	3	1
0.86000	127.196	30	8	4	4
0.83000	136.273	10	10	2	0
0.81000	143.974	20	10	2	2

Deleted By or Rejected By: Deleted by 22-1012
Color: Brownish black to black
Melting Point: 1590
Optical Data: B=2.31

Primary Reference
Publication: Am. J. Sci.
Detail: volume 19, page 67 (1930)
Authors: Poshjak.

**Radiation:**	MoKa	**Filter:**	Not specified
**Wavelength:**	1.54060	**d-spacing:**	
**SS/FOM:**	5.7 (0.097,34)		

Pattern: PDF 81-0681 Radiation: 1.54060 Quality: Calculated

**Formula**	(Zn0.802 Fe0.198) (Fe1.802 Zn0.198 O4)
**Name**	Zinc Iron Oxide
**Name (mineral)**	
**Name (common)**	

**Lattice:**	Cubic	**Mol. weight =**	241.07
**S.G.:**	Fd-3m (227)	**Volume [CD] =**	601.19
		**Dx =**	
		**Dm =**	
		**I/Icor =**	9.570

**a =**	8.43990	**alpha =**	
**b =**		**beta =**	
**c =**		**gamma =**	
**a/b =**	1.00000	**Z =**	8
**c/b =**	1.00000		

ICSD Collection Code: 072036
Remark From ICSD/CSD: REM TEM 298
Remark From ICSD/CSD: REM RVP
Temperature Factor: ITF
Additional Pattern: See PDF 81-672, 81-673, 81-674, 81-675, 81-676, 81-677, 81-678, 81-679, 81-680, 81-682, 81-683, 81-684
Article Title: Temperature dependence of the cation distribution in zinc ferrite (ZnFe2O4) from powder XRD structural refinements

Structure
Publication: Eur. J. Mineral.
Detail: volume 4, page 571 (1992)
Authors: O'Neill, H.S.C.
Primary Reference
Publication: Calculated from ICSD using POWD-12++

**Radiation:**	CuKa1	**Filter:**	Not specified
**Wavelength:**	1.54060	**d-spacing:**	
**SS/FOM:**	999.9 (0.0002,18)		

d	2θ	I	h	k	l
4.87278	18.191	999	1	1	1
2.98396	29.920	169	2	2	0
2.54473	35.240	10	3	1	1
2.43639	36.862	37	2	2	2
2.10997	42.825	78	4	0	0
1.93625	46.885	232	3	3	1
1.72279	53.119	50	4	2	2
1.62426	56.621	3	3	3	3
1.49198	62.168	177	4	4	0
1.42660	65.361	107	5	3	1
1.33447	70.512	16	6	2	0
1.28707	73.524	1	5	3	3
1.27236	74.517	15	6	2	2
1.21819	78.445	8	4	4	4
1.18182	81.354	47	7	1	1
1.12783	86.156	17	6	4	2
1.09878	89.023	2	7	3	1

Pattern: PDF 89-0951 Radiation: 1.54060 Quality: Calculated

**Formula**	Fe3 O4
**Name**	Iron Oxide
**Name (mineral)**	Magnetite
**Name (common)**	

**Lattice:**	Cubic	**Mol. weight =**	231.54
**S.G.:**	Fd-3m (227)	**Volume [CD] =**	612.18
		**Dx =**	
		**Dm =**	
		**I/Icor =**	1.180

**a =**	8.49100	**alpha =**	
**b =**		**beta =**	
**c =**		**gamma =**	
**a/b =**	1.00000	**Z =**	8
**c/b =**	1.00000		

ICSD Collection Code: 085807
Temperature Factor: ATF
Remark From ICSD/CSD: REM TEM 976
Remark From ICSD/CSD: REM NDS
Test From ICSD: At least one TF implausible
Article Title: Refinement of the ferri- and paramagnetic phases of magnetite from neutron multiple diffraction data

Structure
Publication: J. Appl. Crystallogr.
Detail: volume 31, page 718 (1998)
Authors: Mazzocchi, V.L., Parente, C.B.R.
Primary Reference
Publication: Calculated from ICSD using POWD-12++

**Radiation:**	CuKa1	**Filter:**	Not specified
**Wavelength:**	1.54060	**d-spacing:**	
**SS/FOM:**	999.9 (0.0001,18)		

d	2θ	I	h	k	l
4.90228	18.081	134	1	1	1
3.00202	29.736	999	2	2	0
2.56013	35.021	512	3	1	1
2.45114	36.633	28	2	2	2
2.12275	42.554	51	4	0	0
1.94797	46.586	3	3	3	1
1.73322	52.774	18	4	2	2
1.63409	56.250	10	5	1	1
1.50101	61.753	44	4	4	0
1.43524	64.919	1	5	3	1
1.41517	65.956	18	4	4	2
1.34255	70.026	2	6	2	0
1.29487	73.009	1	5	3	3
1.28007	73.993	6	6	2	2
1.22557	77.883	2	4	4	4
1.18898	80.762	1	7	1	1
1.13466	85.513	2	6	4	2
1.10543	88.347	1	5	5	3

Pattern: PDF 21-0920 Radiation: 1.54060 Quality: Blank

Formula Fe2 O3
Name Iron Oxide
Name (mineral)
Name (common)

d	2θ	I	h	k	l
6.01000	14.728	80			
4.36000	20.352	80			
4.15000	21.394	40			
3.60000	24.710	100			
3.00000	29.757	60			
2.74000	32.655	40			
2.40000	37.442	10			
2.00000	45.306	20			
1.87000	48.652	10			
1.81000	50.375	20			
1.58000	58.357	10			

Lattice:
S.G.:

Mol. weight = 159.69
Volume [CD] = 0
Dx =
Dm =
I/Icor =

a =
b =
c =
a/b =
c/b =

alpha =
beta =
gamma =
Z =

Primary Reference
Publication: Bull. Soc. Chim. Fr.
Authors: Walter-Levy, Quemeneur.

Radiation: CuKa1
Wavelength: 1.54060
SS/FOM:

Filter: Not specified
d-spacing:
